# Present and future of liver transplantation for cholangiocellular carcinoma: moving toward personalized multiparametric transplantability patterns

**DOI:** 10.3389/ti.2026.15786

**Published:** 2026-05-07

**Authors:** Umberto Cillo, Alessandro Furlanetto, Jacopo Lanari, Eleonora Nieddu, Eugenia Rosso, Francesco Enrico D’Amico, Domenico Bassi, Enrico Gringeri

**Affiliations:** 1 Department of Surgery, Oncology and Gastroenterology (DiSCOG), University of Padua, Padua, Italy; 2 Hepato-Bilio-Pancreatic Surgery and Liver Transplantation Unit, Azienda Ospedale Universita Padova, Padua, Italy

**Keywords:** cholangiocarcinoma, liver transplant, transplant oncology, transplant assessment, tumor biomarkers

## Abstract

Liver transplantation for cholangiocarcinoma (CCA) shifted from a contraindication to a promising therapeutic option for selected patients. Advances in neoadjuvant therapy and refined selection criteria resulted in long-term outcomes comparable to other accepted oncologic indications, particularly in perihilar CCA managed with standardized protocols and in intrahepatic CCA with favorable tumor biology. The future challenge is to develop a multiparametric biological selection, blending clinical, functional, histopathologic, molecular, and radiologic parameters to identify candidates with indolent disease behavior, thus maximizing oncologic benefit while ensuring appropriate use of limited graft resources.

## Introduction

Liver transplantation (LT) for cholangiocarcinoma (CCA) emerged as a critical area of inquiry due to the limited curative options in a context of a rapidly evolving transplant oncology [1–3]. Although resection remains the standard of care (SOC), many patients are ineligible due to tumor burden, anatomical constraints, or insufficient future liver remnant [4–6]. On a speculative basis, LT may provide a valuable alternative, allowing complete oncologic resection with wide margins, eliminating the pro-oncogenic hepatic microenvironment, and restoring liver function often compromised by underlying disease or prior treatments.

Aside from the excellent outcomes observed in revisited HCC indications, LT is now employed for hepatoblastoma, hemangioendothelioma, and unresectable, well-differentiated neuroendocrine tumors, and selected unresectable colorectal liver metastases patients [7–10]. All these indications share an intrinsic favorable tumor biology. On the contrary, CCA has an aggressive behavior, and LT evolved from a contraindication to a therapeutic possibility only after patient superselection. This review will present current results in the field of LT for CCA, with particular focus on available evidence to improve patient selection based on biological aggressiveness.

## Current landscape of CCA management

CCA is a biologically and clinically heterogeneous malignancy of biliary epithelial cells, characterized by an aggressive course and high recurrence risk. Although rare, its global incidence and mortality increased, ranging from 0.3 to 6/100,000 in Western countries and exceeding 6/100,000 in East Asia, reflecting geographic variability in genetic, environmental, and infectious risk factors [11–13]. CCA is classified anatomically into intrahepatic (iCCA), perihilar (pCCA), and distal (dCCA), each with distinct risk associations: iCCA with chronic liver disease, cirrhosis, viral hepatitis, and obesity; pCCA with primary sclerosing cholangitis; dCCA with choledocholithiasis. Beyond anatomy, CCA shows marked biological heterogeneity in molecular pathogenesis, tumor microenvironment, histology, and growth patterns. Diagnosis is challenging due to asymptomatic early stages and nonspecific imaging. Contrast-enhanced CT is the standard for staging, MRI provides detailed assessment of local and biliary extension, and PET-CT is useful for lymph node and distant staging. Serum CEA and CA19-9 elevation is associated with advanced disease [11, 13–15]. While preoperative histology is not currently required for pCCA due to risk of dissemination, it is recommended that all iCCA candidates for LT undergo liver biopsy to confirm diagnosis, exclude mixed HCC-CCA and to identify poorly differentiated tumors with high risk of recurrence [16–18].

Hepatic resection is considered the main curative treatment for both pCCA and iCCA, with 5-year survival ranging from 25% to 45% [19–21]. Despite innovative and extreme approaches [22, 23], most patients remain ineligible for surgery and can only receive systemic therapies, with median OS not exceeding 12 months [24].

A registry-based study by ENSCCA [19] showed that most favorable outcomes were achieved after radical (R0), node-negative (N0) resection, with a median OS of 52.2 months and a relapse rate of 59.9%. In contrast, patients with positive margins or nodal involvement had 21%–29% 5-year OS, with a 77.4% relapse rate. Resection was performed in only 50.3% of patients, and R0 margin in 35.8%. Among the 49.6% of patients with unresectable disease, median OS was 10.6 months in those treated with active palliative therapy and 4.0 months in those receiving best supportive care.

Gemcitabine/cisplatin (GemCis) has long been first-line therapy for advanced biliary tract cancers [24], but recently the addition of immune checkpoint inhibitors became the new SOC [25, 26]. Despite these developments, the clinical benefit remains modest. In the updated TOPAZ-01 trial [27], durvalumab improved median OS by 1.6 months, while pembrolizumab extended OS by 1.8 months in the KEYNOTE-966 trial [28], compared to Gem-Cis alone. However, the association of Gem-Cis and Durvalumab showed excellent disease control rates (85%), with a 59% rate of sustained response after 6 months, making it a promising candidate as neoadjuvant treatment [25, 29, 30].

These unfavorable outcomes underscore two critical considerations. First, patient selection is crucial, focusing on biological aggressiveness and extrahepatic spread. Performance status, CA19-9, vascular involvement, and tumor size may serve as predictors of futility for both resection and transplantation [31, 32]. Second, although R0 resections are fundamental prerequisites for relevant survival benefit, the risk of recurrence remains high even after oncologically sound interventions. This infers directly to the transplant oncology setting, where total hepatectomy overcomes the problem of positive margins in liver-limited disease, while in cases of direct involvement of adjacent structures pancreaticoduodenectomy or total upper-abdominal exenteration is considered to ensure radicality.

## LT for pCCA

### From early experiences to “standard approaches”

Early reports described dismal outcomes, with 20%–38% 5-year OS and 53%–84% recurrence rates, despite anecdotal cases of long-term survival in early-stage node-negative patients, and a controversial role of primary sclerosing cholangitis (PSC) [33–36].

**FIGURE 1:**
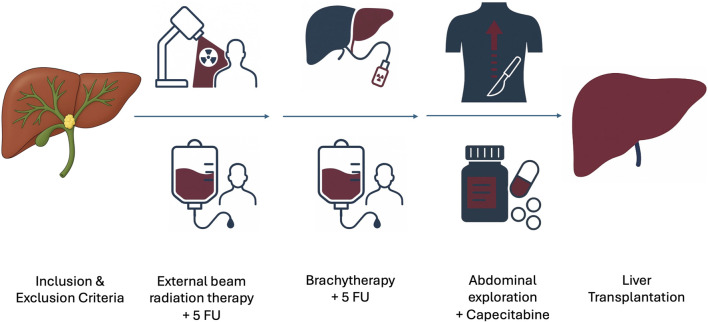
The Mayo Clinic protocol: the neoadjuvant chemoradiation regimen consists of external beam radiation (40–45 Gy), followed by transcatheter brachytherapy (20–30 Gy) using iridium wires. Intravenous 5-fluorouracil is administered concurrently as a radiosensitizer during radiation therapy, and capecitabine-based chemotherapy is given while patients await transplantation. Staging surgery with lymph node biopsies is always performed prior to LT, via laparoscopy, hand-assisted surgery, or laparotomy.

In 1993, the Mayo Clinic [37] described a novel protocol proposing LT for pCCA after thorough selection and aggressive neoadjuvant chemo-radiotherapy (Figure 1; Table 1). The first large case series [38] demonstrated excellent outcomes. Of 184 enrolled patients, 172 completed chemoradiation and underwent staging surgery, and 126 ultimately received LT. The 5-year intention-to-treat (ITT) survival was 54%, reaching 61% in patients with underlying PSC and 42% in those with *de novo* pCCA. Recurrence occurred in 21 patients (18%) after a mean time of 25 months.

**TABLE 1:** Mayo clinic protocol [3, 37–39].

Inclusion criteria	Exclusion criteria
- Diagnosis of pCCA (transcatheter biopsy or brush cytology, CA 19–9 > 100 mg/mL and/or a mass on cross-sectional imaging with a malignant appearing stricture on cholangiography) - Unresectable tumor above cystic duct (pancreatoduodenectomy for microscopic involvement of CBD) or resectable pCCA arising in PSC - Radial tumor diameter <3 cm - Absence of intrahepatic and extrahepatic metastases - Candidate for liver transplantation	- Intrahepatic cholangiocarcinoma - Uncontrolled infection - Prior radiation or chemotherapy - Prior biliary resection or attempt resection - Intrahepatic metastases - Evidence of extrahepatic disease - History of other malignancy within 5 years - Transperitoneal biopsy (including percutaneous and EUS-guided FNA)
Mayo clinic protocol: neoadjuvant chemo-radiotherapy	
- External beam radiation therapy (45 Gy in 30 fractions, 1.5 Gy twice daily) + 5FU for 3 days at initiation - Brachytherapy (20 Gy at 1 cm in approximately 20–25 h) + 5FU - administered 2 weeks following completion of external beam radiation therapy - Capecitabine - administered until the time of transplantation, held during perioperative period for staging - Abdominal exploration for staging - Liver transplantation	

Unresectability is defined as bilateral segmental ductal involvement, encasement of the main portal vein, unilateral segmental ductal involvement with contralateral vascular encasement, or unilateral hepatic atrophy combined with contralateral segmental ductal or vascular involvement, particularly in the presence of underlying liver disease (PSC). Transperitoneal biopsy was introduced as exclusion criteria due to the reported high risk of tumor seeding [40]. As a result, diagnosis of pCCA within this protocol must rely on identification of a malignant-appearing biliary stricture on cholangiography, along with at least one of the following: pathological confirmation by transcatheter biopsy or brush cytology; CA 19–9 level >100 mg/mL; mass visible on cross-sectional imaging; or detection of biliary aneuploidy by fluorescence *in situ* hybridization (FISH) [38].

These findings were confirmed by a multicenter study [41] involving 287 patients from 12 high-volume transplant centers across the USA. In this cohort, 71 patients dropped out before undergoing LT, 5-year ITT survival was 53%, and the recurrence-free survival (RFS) was 65%.

Following these encouraging results several groups in Europe and the US started following the Mayo protocol or Mayo-like protocols with similar inclusion criteria and slight modifications in the neoadjuvant treatment. However, rather small case series were reported.

A long-term analysis from the Mayo Clinic [42] reported 349 patients (1993–2018), of whom only 60% ultimately underwent LT. OS at 5- and 10-year was 69% and 62% in the per-protocol analysis and 51%, and 46% in the ITT analysis. Interestingly, a significant difference in survival was observed between patients with PSC-associated and those with *de novo* pCCA (5-year OS 76% vs. 58%).

A recent meta-analysis [43] of 20 studies comprising 428 patients reported a 31.6% pooled 5-year OS following LT without neoadjuvant therapy, compared to 65.1% in patients who completed neoadjuvant chemoradiation. Furthermore, 3-year recurrence was significantly lower among those who received neoadjuvant therapy (24% vs. 51.7%). However, despite the Mayo Clinic cohort being the largest (152 patients), marked heterogeneity was observed in the application of the protocol across studies. While most studies selected patients with unresectable tumors smaller than 3 cm and excluded those with prior resection or biopsy, only a subset included patients with PSC, elevated CA19-9 levels, or malignant stricture in the absence of positive cytology. Furthermore, some groups (including the Mayo Clinic during its early experience) excluded patients with tumor extension beyond the origin of the cystic duct, to avoid pancreaticoduodenectomy. Although preoperative staging was universally performed, the extent of lymph node sampling varied considerably. Only three studies strictly followed the original Mayo chemoradiation regimen, while others introduced modifications such as substituting 5-fluorouracil with capecitabine or gemcitabine/cisplatin, or omitting chemotherapy or brachytherapy altogether.

In line with these principles, the recent Milan consensus [6] recommended LT only after neoadjuvant treatment with Mayo chemo-radiation regimen, and in presence of an unresectable pCCA <3.0 cm, with no evidence of nodal or distant metastases, no previous surgical manipulation nor transperitoneal biopsy. Interestingly, the jury supports LT also in case of borderline or dubious preoperative resectability.

### Neoadjuvant, multimodal approach or simple patient selection?

The strength of the Mayo Protocol lies in rigorous criteria and locally-aggressive neoadjuvant treatment. However, the relative contribution of these two components to the overall success remains uncertain. An ELITA-ELTR study showed that a subgroup of patients who met Mayo criteria but did not receive neoadjuvant treatment before LT had similar excellent long-term oncological results (5-year OS 59%) and fared significantly better than patient outside criteria (5-year OS 21%) [44].

Although some centers [45], question the utility of neoadjuvant treatment, available data suggest that this approach increases the risk of positive margins and disease recurrence, meanwhile preventing a test of time on biological aggressiveness.

An Italian survey [39] showed that several patients underwent LT for pCCA without receiving neoadjuvant therapy, due to concerns regarding the use of radiotherapy and its short- and long-term complications, along with the requirement to deviate from current SOC chemotherapy.

These concerns are shared by the Mayo Clinic group [46], who, despite reporting excellent outcomes in the per-protocol cohort, also observed a worrisomely high 31% dropout rate (41% for *de novo* and 15% for PSC-associated pCCA). They further highlight the high toxicity of chemoradiation: nearly all patients develop recurrent cholangitis, while vascular friability often results in ischemic cholangiopathy and strictures, frequently progressing to liver failure in the absence of transplantation [38, 42, 47].

Even assuming a therapeutic effect of the neoadjuvant regimen, the considerable dropout rate implies that a several patients who ultimately did not undergo transplantation received suboptimal chemotherapy while being exposed to treatment-related complications, without any survival benefit [45]. It has been argued that radiotherapy (and consequently radiosensitizing fluorouracil) could be avoided unless their role in improving post-transplant outcomes is definitively established, as they are not currently included among standard treatments for advanced pCCA [4, 5].

### The underlying disease issue

Primary sclerosing cholangitis (PSC) is a major predisposing condition for pCCA. Management is particularly challenging due to diffuse biliary involvement, impaired liver function, and a pro-oncogenic field that favors multifocal and synchronous neoplastic transformation [48]. Surgical resection is technically demanding and often associated with high morbidity and incomplete oncologic clearance [49]. When performed according to the Mayo Clinic protocol, LT yields superior outcomes in PSC-associated pCCA compared to *de novo* cases, reflecting earlier diagnosis, less aggressive tumor biology, and the concurrent treatment of both the malignancy and the underlying cholangiopathy [42, 43]. Reported results show 5-year overall survival of 65%–70% and recurrence rates of 20%–24%, making neoadjuvant therapy and LT the treatment of choice in candidable patients with PSC-associated pCCA [48].

### Role of pancreaticoduodenectomy

Hepatopancreatoduodenectomy (HPD) is an extremely complex and technically demanding procedure associated with high morbidity. The technique has been mainly developed and reported by Japanese groups [50–54], who also provided most of the available outcome data [52, 55–59]. A recent meta-analysis [60] reports a 90-day mortality of 10% and morbidity of 64%, although mortality can approach zero in highly experienced centers [61]. Outcomes show marked geographic variability, with 90-day mortality of 26% in North America [62], 13%–17% in Europe [63], and <5% in Japan [58, 61].

The combination of total hepatectomy, pancreaticoduodenectomy (PD) and LT has been poorly explored. PD may be performed simultaneously with transplantation or delayed by weeks to months, but evidence is limited to small series and case reports [35, 42, 64–68], with long-term survival mainly driven by CCA recurrence [64]. The addition of pancreaticoduodenectomy increases morbidity, particularly due to technical complexity and to the impact of immunosuppression on pancreatic healing. Pancreatic fistula, reported in up to 24% of cases [64], is especially critical in the transplant setting because of the risk of vascular anastomotic injury or compression; total pancreatectomy or a two-stage approach may be considered in case of complications.

### Living donor liver transplantation (LDLT)

Although potentially advantageous for optimizing transplant timing in the neoadjuvant setting, LDLT was limited by concerns regarding the risk of arterial thrombosis related to perihilar irradiation. Although jump-grafts to the aorta can be used, and the middle-colic or right gastroepiploic artery were employed to avoid performing anastomoses in the irradiated field, these strategies remain technically demanding [69, 70]. Recent neoadjuvant protocols that omit pre-transplant radiotherapy [39, 71, 72] have renewed interest in the use of LDLT for pCCA.

A retrospective analysis [73] by the Mayo Clinic compared 73 cases of LDLT performed for pCCA (66% PSC-associated) with 173 LDLTs for other indications. The pCCA group showed higher requirement for arterial or portal vein reconstruction and Roux-en-Y choledochojejunostomy. Rates of early hepatic artery thrombosis were similar between the two groups (5.4% vs. 7.6%), whereas late arterial (18.9% vs. 4.1%) and portal (37.8% vs. 8.7%) complications were more frequent in the pCCA group, although these did not affect long-term survival. 5-year OS was significantly lower in the overall pCCA cohort (66.5% vs. 87%), and differed between *de novo* (47.5%) and PSC-associated (75.9%) cases. The Mayo Clinic tried to address the issue of operating in an irradiated field by introducing technical modifications, particularly within LDLT protocols [73]. These include nonstandard arterial reconstruction (avoiding irradiated hepatic artery, use alternative inflow sources with interposition grafts, anastomosis to the infrarenal or supraceliac), portal reconstruction (using jump grafts or anastomosis to the superior mesenteric vein or splenic vein confluence below the irradiated field) and systematic biliary reconstruction with Roux-en-Y choledochojejunostomy [42, 47].

The University of Kyoto [70] drafted a modified protocol for LDLT in pCCA, consisting of GCS chemotherapy administered for more than 2 months, followed by external-beam radiotherapy only in case of disease stability. In their initial report on 10 patients, only five proceeded to LT, achieving 100% 1-year survival rate, with one recurrence after 10 months. Hepatic artery thrombosis and delayed gastric emptying occurred in two and three patients, respectively.

### Comparing resection and transplantation

The excellent long-term outcomes after LT, contrasting with persistently poor results after liver resection, raised the issue whether LT should be extended beyond unresectable disease to include borderline-resectable or even resectable cases. Only few studies addressed this issue, and case series are small and heterogeneous. A 2019 systematic review and metanalysis [74] of studies comparing LT and LR suggested a trend towards longer OS after LT, although not statistically significant. Their analysis, however, showed comparable mortality rates, but shorter hospital stay and higher rates of R0 margins after LT. In contrast, the most recent report from the Mayo Clinic [46] focusing on *de novo* pCCA, demonstrated superior results of LT compared with resection (with or without vascular resection) in terms of OS (78 vs. 25.8 vs. 58.2 months) and perioperative mortality (4% vs. 8% vs. 7%) in the per-protocol analysis. However, the high dropout rate (31% in the LT group, 28% in the surgical group) had a substantial impact on the ITT analysis, which failed to demonstrate a significant survival advantage of LT over resection.

Dropout rate is a crucial and underestimated factor. The randomized TRANSPHIL trial (NCT02232932), comparing neoadjuvant chemoradiation and LT with liver resection for resectable pCCA, reported poor long-term survival and a dropout rate exceeding 50%, ultimately leading to early termination for ethical reasons. Exposing resectable patients to both the toxicity of chemoradiation and the high likelihood of dropout and futility may ultimately condemn them to poor outcomes associated with chemotherapy alone, rather than the still unfavorable but comparatively better results of resection. These findings warrant caution and at the same time support efforts to improve consistency in preoperative management, favoring SOC chemotherapy over chemoradiation [39].

### Benchmarking surgical therapeutic options

A benchmark study [75] involving 134 patients from 17 high-volume centers provided several important insights. Ideal cases were defined as treated at high-volume centers (≥50 LT/year), who underwent neoadjuvant chemoradiotherapy, had tumors <3 cm, negative lymph nodes, and no significant comorbidities. Benchmark thresholds included 90-day mortality rate ≤5.2%, 1-year Comprehensive Complication Index (CCI) ≤33.7, ≤66.7% grade ≥3 complications, and R0 resection margins rate ≥80.0%. For long-term outcomes, the benchmarks for 5-year disease-free survival (DFS) and OS were ≥43.8% and ≥60.0%, respectively. Authors advocate for recognizing unresectable pCCA treated with neoadjuvant chemoradiotherapy as a formal indication for LT, and propose extending its use to resectable cases based on the observation that benchmark outcomes of LT for pCCA not only exceed those of LT for other indications [76] but also those of surgical resection [21]. In this study, benchmark LT cases were also directly compared with a matched cohort of curatively resected, node-negative Bismuth IV patients, demonstrating significantly superior 5-year DFS (50.2% vs. 17.4%) and OS (56.3% vs. 39.9%) in the LT group, with no significant difference in major complications (72.7% vs. 74.6%) and a higher 3-month mortality rate in the resection group.

### Future directions

**FIGURE 2:**
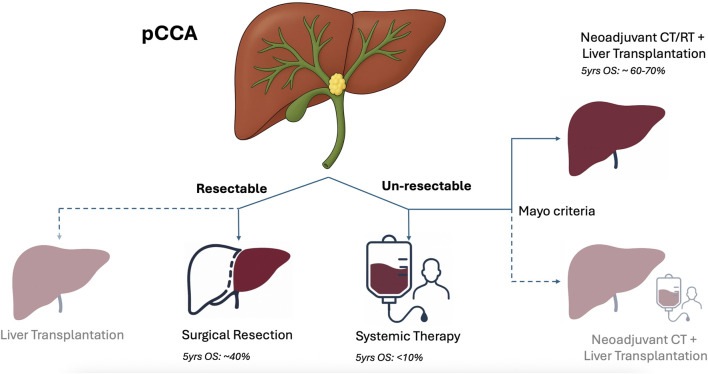
Current landscape of pCCA treatment (shaded indications are investigational).

To address the limitations of the Mayo protocol, several groups shifted to a neoadjuvant treatment based on SOC chemotherapy, with various adoption of radiotherapy or transarterial radioembolization (Figure 2).

An undergoing Italian trial (LITALHICA, NCT06125769) maintains Mayo selection criteria but replaces neoadjuvant radiochemotherapy with SOC chemotherapy to avoid altering the patient’s therapeutic pathway solely due to trial inclusion [77].

Ongoing trials are summarized in Table 2, the main clinical studies and their key outcomes are reported in Table 3, and the main statements with supporting studies, corresponding levels of evidence, and relevant guideline recommendations are provided in Supplementary Table 1.

**TABLE 2:** LT for pCCA ongoing trials.

Study	Study title	Inclusion criteria	Neoadjuvant treatment	Outcomes	Center	Start date
NCT01549795	Liver transplantation for hilar cholangiocarcinoma in association with neoadjuvant radio- and chemo-therapy	UpCCA<3 cm, PSC, no prior chemotherapy or surgery	Radiotherapy + brachytherapy + caepcitabine	Recurrence rate, time to recurrence, DFS, OS, morbidity	Padova University Hospital, Padova (Italy)	2012
NCT02178280	Phase 1 study of liver transplantation combined with neoadjuvant radiochemotherapy for unresectable hilar cholangiocarcinoma	UpCCA <3 cm, N0 M0, <65yo	Brachytherapy (I-125 stents) followed by external beam radiotherapy + capecitabine	OS, RFS, acute and chronic rejection rate	The Affiliated Nanjing Drum Tower Hospital of Nanjing University Medical School, Nanjing (China)	2014
NCT04378023	Liver transplant combined with neoadjuvant chemo-radiotherapy in the treatment of unresectable hilar cholangiocarcinoma. A prospective multicenter study	UpCCA, <3 cm, N0 M0, no prior surgery, <70 yo	External radiotherapy + capecitabine, followed by gemcitabine + cisplatin	OS, RFS, ITT OS, drop out rate	Hospital Vall d'Hebron, Barcelona (Spain)	2020
TESLA II (NCT04993131)	Liver transplantation for non-resectable perihilar cholangiocarcinoma	UpCCA (even with portal or arterial infiltration), N0 M0, 6 months SD or PR, 12 months from diagnosis	Chemotherapy	OS, OS after recurrence, DFS, morbidity, QoL	Oslo University Hospital, Oslo (Norway)	2021
LITALHICA (NCT06125769)	LIver TrAnspLantation for non-resectable Peri-HIlar cholangioCArcinoma (LITALHICA)	UpCCA <3 cm, N0 M0, 6 months SD or PR, no prior surgery or biopsy, <70 yo	SOC chemotherapy	OS, DFS, drop out, QoL, patient stratification, role of PET-MRI	Padova University hospital, Padova (Italy)	2024
EMPHATIC (NCT06434493)	Evaluation of combined Modality Protons and hepatic transplantation for hilar cholangiocarcinoma	PSC, UpCCA<3 cm, N0 M0, no prior surgery or radiation	Proton beam therapy (PBT) + capecitabine, followed by chemotherapy (GemCis)	Toxicity, rate of LT, morbidity, cancer-related mortality, graft survival, OS, RFS, recurrence, recruitment rates	University College London Hospitals, London (UK)	2024
SURE-LT (NCT06850753)	​	UpCCA beyond Mayo clinic criteria (including arterial and portal infiltration), M0 (including distant lymph nodes); pCCA recurrence in PSC 2 years after resection (N0R0). 6 months SD or PR	Chemotherapy + radiation followed by en bloc resection of the liver and Pancreas with a “non-touch” technique	OS (1, 3, 5 years), DFS, survival after recurrence, QoL, morbidity	Oslo University Hospital, Oslo (Norway)	2025

UpCCA, unresectable perihilar cholangiocarcinoma; PSC, primary sclerosing cholangitis; SD, stable disease; SOC, standard of care; OS, overall survival; DFS, disease-free survival; RFS, recurrence-free survival; ITT, intention to treat; PR, partial response; QoL, quality of life.

**TABLE 3:** results from key studies about LT for pCCA.

Authors	Study design	Population	N	Key findings	Survival	Main prognostic factors
Meyer et al. [33]	Retrospective study Multicenter 1968–1997	LT for CCA	207	High rate recurrence. LT is not the standard. Neoadjuvant therapy are necessary for LT implementation	1-, 2-, and 5-year OS 72, 48, and 23%; recurrence 51%, 84% recurrence within 2 years. Survival after recurrence rarely more than 1 year	Survival: Tumor recurrence Recurrence; tumor spread at time of surgery
Robles et al. [34]	Multicenter retrospective (Spain) 1988–2001	LT for iCCA/pCCA	36	LT has favourable outcomes, especially with early stage tumors. High selection of patients is required	1-, 3-, and 5-year OS 82%, 53%, and 30%	Survival: Lymphnodes involvement, metastatic disease, advanced stage, vascular invasion, perineural invasion
Heimbach et al. [37]	Prospectic single center 1993–2003	Mayo clinic protocol LT for pCCA	56	Neoadjuvant CRT before LT is essential in LT protocol for pCCA.	1- and 5-year OS = 88% and 82%	​
Ghali et al. [36]	Retrospective single center 1996–2003	LT for incidental iCCA/pCCA	10	Outcomes for transplanted incidental CCA are not better than known CCA. Aggressive investigation pre LT is mandatory	Recurrence in 8/10 patients, 7/10 died because of recurrence. mRFS = 26 months, mOS = 30 months. 3-year OS = 30%	​
Heimbach et al. [78]	Prospectic single center 1993–2006	Mayo clinic protocol LT for pCCA	65	Older patients and those with high CA-19.9 levels, and larger tumors are more likely to develop recurrent disease. Prolonged waiting time may emerge as a significant risk factor	5 years OS 76%, DFS 60%	Predictors of recurrence: older age, pretransplant cancer antigen (CA) 19–9,100 U/mL, prior cholecystectomy, mass on cross-sectional imaging, residual tumor in explant 2 cm, tumor grade and perineural invasion in explant
Seehofer et al. [35]	Retrospective single center/cohort study 1992–2007	LT and extended bile duct resection for pCCA	16	Extended surgical procedures in combination with LT are related to significantly increased perioperative mortality. Adjuvant or neoadjuvant therapy protocol are required to improve outcomes after LT.	1-, 5-, and 10-year OS rates after EBDR + LT 63%, 38%, and 38%	Survival: Metastatic disease, positive lymph nodes, CA19-9 levels >1000, preoperative PTCD (instead of ERCP)
Darwish Murad et al. [41]	Multricenter (12 centers) retrospective study 1993–2010	Mayo clinic protocol LT for pCCA	287	Neoadjuvant CRT is highly effective in LT protocol for CCA. There is a variability in neoadjuvant protocols (variable administration of brachytherapy). Strict patient selection is recommended	2- and 5-year Intent-to-treat = 68% and 53%. 2- and 5-year RFS after LT rates = 78% and 65%	Recurrence: metastatic disease, tumor size >3 cm, direct tumor biopsy, other malignancy in the previous 5 years
Darwish Murad et al. [79]	Multricenter retrospective study 1993–2010	Mayo clinic protocol LT for pCCA	199 (137 LT)	Risk of dropout is related to patient and tumor characteristics. Recurrence risk is mostly associated with presence of residual cancer on explant. PSC patients do not have an Independent survival advantage over *de novo* patients, but present with more favorable tumor Characteristics	​	Predictors of dropout: CA 19–9≥ 500 U/mL, mass ≥3 cm, malignant brushing or biopsy and MELD score ≥20 Predictors of recurrence: Elevated CA 19–9, portal vein encasement and residual tumor on explant
Croome et al. [80]	Retrospective single center (Mayo) 1993–2013	LT vs. LR for pCCA	LT 90, LR 124	Patients with clearly resectable *de novo* pCCA should be treated with LR because there is no evidence that they would fare better with LT.	1-, 3-, and 5-year OS 90%, 71%, and 59% for LTX and 81%, 53%, and 36% for LR. Survival was not different after adjusting for prognostic factors	Survival: Resection (vs. transplantation) age, lymph node metastases, tumor grade and tumor size
Mantel et al. [44]	Multicenter retrospective (ELTR 21 centers) 1990–2010	LT for pCCA	147	LT for pCCA has favourable outcomes with strict selection of patients, according to Mayo clinic criteria	5-year OS after LT for pCCA = 32%. 5-year OS in stricted selected patients (Mayo clinic criteria) = 59%. 90-day mortality rate = 15%. 5-year recurrence probability in stricted selected patients = 46%, in not selected patients 79%	Survival: Lymphnodes involvement
Ethun et al. [81]	Multicenter (USEBMC database) 10 centers (USA) 2000–2015	LT for pCCA	LR 234, LT 70	LR for pCCA that meets criteria for LT (<3 cm, lymph-node negative disease) is associated with substantially decreased survival compared to LT for the same criteria with unresectable disease	OS LT vs. LR 3-yr: 72%vs33%; 5-yr: 64% vs. 18% (p < 0.001); for tumors <3 cm, n-, non PSC, OS LT vs. LR 3-yr: 54%vs44%; 5-yr: 54% vs. 29% (p = 0.03)	​
Moris et al. [74]	Meta-analysis (5 studies)	LT vs. resection for pCCA	​	OS is not inferior after LT in non metastatic unresectable tumors compared to LR. No differences in post operative mortality. Trend towards better OS in LT	​	​
Tan et al. [73]	Retrospective LDLT study Single center 2000–2017	LDLT for pCCA (Mayo clinic)	74	The incidence of early hepatic artery thromboses was similar in LT for pCCA and non-pCCA patients. Late hepatic artery and portal vein complication were more common in the pCCA group	1-, 5- and 10- year OS = 84.9%, 66.5%, and 55.6%. Cancer recurred in 12.3%	Survival: *de novo* pCCA (vs. PSC-associated pCCA), residual tumor
Zaborowski et al. [82]	Prospective single-center (Ireland) 2004–2016	LT for pCCA after NCR	37 ITT (26 LT)	NCR followed by LT substantially increases the survival of patients with unresectable pCCA. Achieving a pathologic complete response confers a significant survival benefit	Overall median survival was 53 months and 1-, 3-, and 5-year OS was 81%, 69%, and 55%	Survival: Complete response
Cambridge et al. [43]	Meta-analysis (20 studies) 2000–2019	LT for pCCA	428	Better OS in LT for pCCA after completed NCT. Better results in LT for PSC-associated pCCA compared to pCCA alone	1, 3-, and 5-year OS rates after LT = without NCT 71.2%, 48.0%, and 31.6%; with NCT 82.8%, 65.5%, and 65.1%. 3-year RFS = 24.1% with NCT and 51.7% without NCR therapy	​
Breuer et al. [75]	Benchmark study, multicenter (17 centers) 2014–2018	LT for pCCA (Mayo-like protocol, tumor 3 cm, node-negative)	134	NCT + LT for pCCA must be considered in selected patients with unresectable tumor (negative nodes and size < 3 cm). LT should be considered also in selected resectable patients, even considering LDLT.	Benchmark 5 years OS >60% DFS >48.3%; superior compared with a matched group of nodal negative patients undergoing LR	​
Hoogwater et al. [45]	Multicenter retrospective, cohort study 2011–2020	LT for pCCA	49	NCT before LT is related to a higher complication rate (vascular), higher survival rate and lower recurrence rate after LT.	1-, 3-, and 5-year OS after LT with NCT = 65%, 51% and 41%; after LT without NCT = 91%, 68% and 53%. Hepatic vascular complications are more frequent after NCT	Recurrence: neoadjuvant therapy before LT, patients BMI, tumor size in final pathology, vascular invasion, perineural invasion
Dong et al. [46]	Retrospective cohort study Single center 1993–2023	LT (Mayo protocol) vs. liver resection (with or w/o vascular reconstruction) for pCCA	191	NCT + LT offers best outcomes for unresectable patients. LR + VR remains the preferred approach for resectable patients. Key factors are high drop out rates in LT and high perioperative mortality after LR.	Matched cohorts: 5-year OS rate in LR w/o VR = 60.8%, in RT + LT = 44.2% and LR + VR = 23.6%. Median RFS in RT + LT = 46.7 months, in LR w/o VR = 32.3 months, in LR + VR = 17.7 months. After matching the LR w/o VR group remained the most favorable group with the highest RFS, followed by RT + LT and LR + VR	​
Ito et al. [70]	Prospective single-center 2018–2024	LDLT after CRT for pCCA	10 (5 LDLT)	LDLT for pCCA is feasible and effective but it is the last treatment option	1- and 5-year OS 100%, 27.4%. High frequency of HAT	​

## LT for iCCA

Historically, LT for iCCA was associated with poor outcomes (10%–18% 5-year OS) and high recurrence. However, recent developments identified two subsets of patients with potential high transplant benefit [16]:

1. Cirrhotic patients with unresectable (due to impaired liver function) “very early stage” iCCA (single tumor, ≤2 cm)
2. Locally advanced iCCA after good response to neoadjuvant chemotherapy

### LT for “very early” iCCA in cirrhosis

In cirrhotic patients with severe portal hypertension and small unresectable iCCA, LT may simultaneously treat the tumor and the chronic liver disease.

A 2014 Spanish retrospective study [83] reported 62% 5-year OS after transplantation among cirrhotic patients with small incidental iCCA. A subsequent international study [84] showed that similarly defined “very early” iCCA (<2 cm) had better outcomes compared to “advanced (multiple or >2 cm) tumors (5-year OS 65% vs. 45%). Risk of recurrence at 5 years was also lower in the very early group (18% vs. 65%), although tumor size was not a predictor of tumor recurrence at multivariate analysis.

A metanalysis [85] of 18 studies including 355 cases, showed that cirrhosis, was positively associated with RFS, and at subgroup analysis patients with very early iCCA had superior pooled 5-year RFS compared to advanced iCCA (67% vs. 34%). To be noticed, incidental diagnosis was not associated with either prolonged OS or RFS.

However, real life applicability of the 2 cm cutoff may be difficult, as pre-transplant confirmation such small unresectable iCCA is quite uncommon. Both HCC and CCA can develop on cirrhosis, as long as mixed HCC-CCA forms, and preoperative differential diagnosis can be challenging [86–88]. Indeed, a prospective trial on LT for early iCCA (NCT02878473) by the Toronto group was terminated because of low accrual.

### LT for locally advanced iCCA after neoadjuvant chemotherapy

Attaining R0 resection for locally advanced iCCA can be challenging even in the non-cirrhotic [20]. To this respect, total hepatectomy followed by LT represents a resection with the highest potential for radicality, provided that there are no lymphnode involvement and extrahepatic spread. Evidence suggest, however, that patients should be selected based on surrogates of favorable tumor biology, namely response to neoadjuvant chemotherapy and test of time of disease control.

The group from UCLA [89, 90] in a 24-year single center experience on 35 cases, highlighted how patients receiving LT had significantly better outcomes than those receiving resection (5-year RFS 33% vs. 0%). Moreover, in the LT group, patients receiving neoadjuvant and adjuvant chemotherapy had better survival compared to those receiving no therapy or adjuvant therapy alone (5-year RFS 47% vs. 20% vs. 33%). On multivariate analysis, recurrence was not associated with tumor size, but rather with factors biology-related factors like multifocality, infiltrative pattern, pernieural and lymphovascular invasion, history of PSC, neoadjuvant and adjuvant therapy. In 2022, the same group reported their 30-year experience [91] (19 pCCA and 30 iCCA), confirming excellent oncological results for LT even for large size CCAs compared to patients not receiving preoperative treatment, particularly when adopting a multimodal chemotherapy and loco-regional neoadjuvant approach (5-year OS 100% vs. 41%).

The Houston Methodist-MD Anderson group developed a protocol offering LT to patients with unresectable iCCA, without evidence of macrovascular or lymph node involvement, who had sustained tumor stability with gemcitabine-based neoadjuvant therapy for more than 6 months [92]. Their latest series [93] (18 patients) showed post-LT OS of 71%, and 57% at 3 and 5 years respectively. Tumor recurred in 39% of patients after a median time of 11 months after LT, being treated with further systemic therapy and surgery. Interestingly, transplanted patients had a median number of 2 iCCA tumors and a median cumulative tumor diameter of 10.4 cm, confirming that acceptable OS can be achieved independently from size in presence of good response to therapy and disease stability. Next-generation sequencing was performed in most cases, using liquid biopsy, percutaneous biopsy, or explant tumor tissue. Known genetic alterations were identified, including FGFR (27%), CDKN2A (7%), IDH1 (35%), BRAF (19%), and TP53 (19%), but univariate analysis showed no association with outcomes. In selected patients, the presence of targetable alterations enabled the use of targeted therapies, including the FGFR inhibitor pemigatinib (1 case), the IDH1 inhibitor ivosidenib (2 cases), and the PARP inhibitor olaparib (4 cases).

### LDLT in iCCA

Patients with iCCA were traditionally excluded from LDLT because of insufficient expected OS and RFS to justify the donor’s risk. However, the evolving diffusion of the concept of transplant benefit as gain in life-years quality-adjusted over alternative available therapies is now changing such a perception, provided the achievement of a minimal 5-year survival to avoid futility.

Literature remains limited [94, 95]. A multicenter study from Japan [96] on 19 LDLT recipients incidentally diagnosed with iCCA showed 46% 5-year OS. A similar study from Pakistan [97] including patients with incidental pCCA and iCCA, collected 16 patients, reporting 47% 3-year RFS (64% for well differentiated tumors). The largest series [98], focusing on LDLT outcomes for primary sclerosing cholangitis, included 55 out of 805 cases with iCCA, with OS reaching 81.9%.

The 2024 ILTS–ILCA Consensus [16] recommends considering LDLT for iCCA within institutional study protocols, particularly for patients with early-stage disease. At the same time, the Toronto group is currently leading a multicentric trial (NCT04195503) to validate LDLT’s efficacy for advanced iCCA.

### Comparative efficacy of resection versus transplantation

Early reports showed markedly worse survival after LT compared with resection [99, 100], although patient matching was frequently biased [94].

A propensity score–matched (PSM) analysis by Jung et al., (16 LT vs. 100 resections), showed comparable OS and recurrence rates [101]. Several groups subsequently analyzed data from the US-NCDB [102–105], consistently reporting similar OS between LT and resection. However, in a multivariate analysis [104], LT was associated with a significantly reduced risk of death compared with matched resection cases.

Huang [106] recently analyzed the US-SEER database, including 2538 patients with iCCA treated with curative surgery (2425 resections, 113 LT) and 5048 LT for HCC. PSM between resected and transplanted iCCA groups corrected the baseline imbalance, since patients with early stage, smaller tumors, well-differentiated histology, and cirrhosis were more likely to be selected for LT. LT patients had significantly longer survival than those who underwent LR in the matched cohorts (median OS: 23 vs. 18 months; 5-year OS 52.8% vs. 29.9%). Interestingly, a subgroup analysis showed that patients who met recommended selection criteria (i.e. very early iCCA on cirrhosis or locally advanced iCCA after chemotherapy) had a 5-year OS of 43.8% and 61.7% respectively.

### Future directions

**FIGURE 3:**
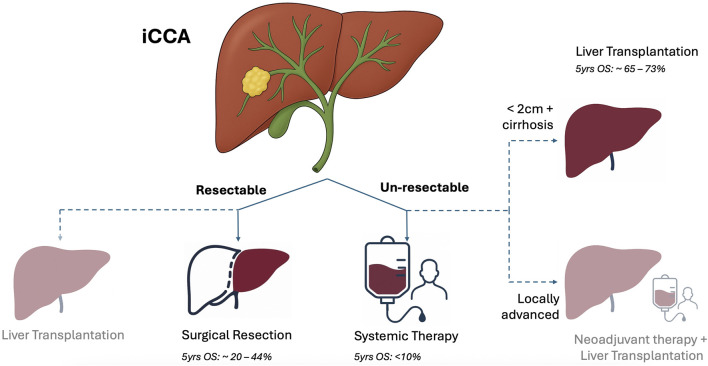
Current landscape of iCCA treatment (shaded indications are investigational).

Preliminary data [107] from the TESLA trial report 5 patients showing excellent perioperative course after LT after neoadjuvant treatment, although two experienced disease recurrence within 12 months (Figure 3; Table 4).

**TABLE 4:** LT for iCCA ongoing trials.

Study	Study title	Inclusion criteria	Neoadjuvant treatment	Outcomes	Center	Start date
NCT04195503	Liver transplant for stable, advanced intrahepatic cholangiocarcinoma	UiCCA, N0 M0, 6 months SD - LDLT	6 months SOC chemotherapy	OS, DFS (5 years)	University Health Network, Toronto (Canada)	2019
TESLA trial (NCT04556214)	Liver transplantation for non-resectable intrahepatic cholangiocarcinoma: a prospective Exploratory trial	UiCCA, N0 M0, 6 months SD	Chemotherapy or locoregional therapy	OS, DFS, morbidity, QoL, retransplantation	Oslo University Hospital, Oslo (Norway)	2020
NCT06140134	Liver transplantation in intrahepatic cholangiocarcinoma	UiCCA, N0 M0, 6 months SD	Systemic therapy	OS, RFS, ITT OS, morbidity	State University of New Jersey, Newark (USA)	2023
LIRICA (NCT06098547)	LIver transplantation for non-resectable intrahepatic CholAngiocarcinoma (LIRICA)	UiCCA, N0 M0, 6 months SD or PR	SOC chemotherapy	OS, DFS, drop out, QoL, patient stratification, role of PET-MRI	Padova University Hospital, Padova (Italy)	2024
LIVINCA (NCT06539377)	Living donor liver transplantation for intrahepatic cholangiocarcinoma	UiCCA, G1-2, M0, SD or PR afetrr neoadj therpay, LDLT	Any chemotherapy regime + mandatory local-ablative therapy (SIRT)	OS, RFS, donor and recipient morbidity	Jena University Hospital, Jena (Germany)	2024
RIS-TH (NCT06910722)	Liver transplantation for locally advanced intrahepatic cholangiocarcinoma after SIRT and chemotherapy	Pauci nodular (≤5 lesions) UiCCA M0, infiltration <50% of liver, <65 yo	Selective internal radiation therapy (SIRT) + chemotherapy	OS (3 years), drop out, recurrence, tolerance, QoL, complications	Assistance Publique - Hôpitaux de Paris, Paris (France)	2025
iCOLA (NCT06862934)	Liver transplantation for unresectable intrahepatic colangiocarcinoma after sustained response to neoadjuvant treatments	UiCCA, N0 M0, 6 months SD or PR	Chemotherapy+/-immunotherapy and transarterial radioembolization (Y90-TARE)	OS, RFS, morbidity, QoL, comparison with resectable patients	Istituto Nazionale dei Tumori, Milan (Italy)	2025

UiCCA, unresectable intrahepatic cholangiocarcinoma; SD, stable disease; LDLT, living donor liver transplantation; SOC, standard of care; OS, overall survival; DFS, disease-free survival; RFS, recurrence-free survival; ITT, intention to treat; PR, partial response; SIRT, Selective Internal Radiation Therapy; QoL, quality of life.

In Italy, the LIRICA trial, is enrolling patients with unresectable iCCA after 6 months of SOC chemotherapy. As for LITALHICA trial, unresectability is assessed by a dedicated multidisciplinary tumor board and patients are listed for LT only after 6 months of documented disease stability.

The Milan-INT group is investigating the neoadjuvant combination of chemotherapy and transarterial radioembolization (Y90-TARE). Preliminary data from 13 patients revealed a 69% dropout rate due to disease progression or inadequate response. Only four patients proceeded to transplantation, showing favorable early outcomes [108].

Ongoing trials are summarized in Table 4, the main clinical studies and their key outcomes are reported in Figure 3 and Table 5, and the main statements with supporting studies, corresponding levels of evidence, and relevant guideline recommendations are provided in Supplementary Table 2.

**TABLE 5:** results from key studies about LT for iCCA.

Authors	Study design	Population	N	Key findings	Survival	Prognostic factors
Pichlmayr et al. [99]	Retrospective study (historical) Single center 1980–1993	LT and LR for iCCA	18 LT	LR is indicated in resectable situations. LT for unresectable lesions seems not to be indicated unless adjuvant protocols appear promising	mOS = 12.8 months after LR, = 5.0 months after LT. Longest survival after transplantation was 25 months. After LR 4 patients survived >5 years. 1-year OS = 13.9% after LT.	Surival: Tumor size, tumor stage
Weimann et al. [100]	Retrospective cohort study Single center 1978–1996	LT and LR for CCA	162 (24 LT)	Therapeutic efforts should therefore be directed towards achieving resectability. Data rule out LT as a treatment option for advanced unresectable CCC	1-, 2- and 5-year survival rates after LR (resectable tumors) = 64%, 43% and 21%, after LT = 21%, 8% and 0%	Survival: Age, jaundice, N and M category, and UICC tumour stage (tumor number, tumor size, treatment modality, vascular invasion, CEA)
Hong et al. [89]	Single-center retrospective Single center (UCLA) 1985–2009	LT vs. resection in locally advanced iCCA/pCCA	57 (38 LT, 19 LR)	LT associated with neoadjuvant and adjuvant therapies is superior to LR with adjuvant therapy in locally advanced iCCA and pCCA	5-year RFS 33% after LT. 5-year OS after neoadjuvant and adjuvant therapies = 47%, with no therapy 20%, with adjuvant therapy only 33%	Survival: hilar CCA (vs. intrahepatic), multifocal tumors, perineural invasion, treatment modality (resection vs. LT), adjuvant and/or neoadjuvant therapy
Hong et al. [90]	Single-center retrospective 1985–2010	Recurrence after LT for iCCA/pCCA	40	Model highly predictive of long term outcomes according to risk stratification after LT for locally advanced iCCA and pCCA	5-year RFS was significantly higher in low-risk (78%) compared with intermediate- (19%) and high-risk (0%) groups	Recurrence: Multifocal tumor, perineural invasion, infiltrative growth pattern, lack of neoadjuvant and adjuvant therapy, history of primary, and sclerosing cholangitis
Sapisochin et al. [83]	Multicenter retrospective cohort study 16 centers (Spain) 2000–2010	LT for incidental CCA/HCC-CC	42	Patients with uninodular tumors 2 cm or smaller had similar OS compared to HCC	Patients with uninodular tumors 2 cm or smaller had similar 1-, 3-, and 5-year survival rate (92%, 83%, 62% vs. 100%, 80%, 80%; P = 0.4)	​
Sapisochin et al.2016 [84]	Multicenter retrospective cohort study 17 centers 2000–2013	LT for very early CCA (incidental or not)	81	Favorable long-term survival after LT for very early intrahepatic cholangiocarcinoma (≤2 cm)	1-, 3-, and 5-year recurrence risk 7%, 18%, and 18% in very early iCCA, 30%, 47%, and 61% in advanced iCCA. 1-, 3-, and 5-year OS 93%, 84%, and 65% in very early iCCA, 79%, 50%, and 45% in advanced iCCA	Recurrence: Microvascular invasion, poor differentiation, tumor size, advanced stage, out of UCSF criteria
Jung et al. [101]	Retrospective cohort study Single center 2003–2014	LT and LR for incidental iCCA	16 LT, 100 LR	The prognosis of incidentally detected ICC following LT is as poor as that following LR.	1-, 3-, and 5-year recurrence rates = 56.2%, 56.2%, and 78.1%. 1-, 3-, and 5-year OS rates were 81.3%, 52.4%, and 52.4%	​
Lunsford et al. [92]	Prospective case series Single center (MD Anderson) 2010–2017	Neoadjuvant therapy followed by LT for locally advanced iCCA	6	Selected patients with locally advanced iCCA who show pre-transplant disease stability on neoadjuvant therapy might benefit from liver transplantation	1-, 3-, 5-year OS = 100%, 83.3%, and 83.3%. Median RFS of 7.6 months after LT. 1-, 3- and 5-year RFS = 50%	​
De Martin et al. [88]	Multicenter retrospective cohort study 3 centers (France) 2002–2015	LT vs. LR for CCA in cirrhosis	75	LT may offer a benefit for highly selected patients with cirrhosis and unresectable iCCA/cHCC-CCA having tumors ≤5 cm	5-year RFS = 75%, 5-year OS = 69% in patients with tumors ≤2 cm and and 65% in patients with tumors >2–5 cm	Recurrence: Tumor size, tumor differentiation, resection (vs. LT)
Ziogas et al. [85]	Meta-analysis 18 studies	LT for iCCA	355	Cirrhotics with very early iCCA or carefully selected patients with advanced iCCA after neoadjuvant therapy may benefit from LT	1-, 3-, and 5-year OS rates = 75%, 56%, and 42%. 1-, 3-, and 5-year RFS rates = 70%, 49%, and 38%. Recurrence rate = 43%	Recurrence: Cirrhosis (protective)
Hara et al. [96]	Multicenter retrospective 45 centers (Japan) 2001–2015	Incidental iCCA in LDLT	19	Incidental iCCA at LT is associated with a high risk of recurrence and poor prognosis	1-, 3-, and 5-year RFS rates = 79%, 45%, and 45%. Tumor recurrence after LT = 53%. 1-, 3-, and 5-year OS rates = 79%, 63%, and 46%	​
Hue et al. [102]	Retrospective registry study cohort Multicenter (national cancer database) 2010–2016	LT and LR for incidental iCCA	1879 LR, 74 LT	LR and LT were associated with similar postoperative outcomes and survival. Hepatectomy is preferable for localized ICC.	1-, 3-, 5-year OS after LT = 89.4%, 53.0,% 40.8%. 1-, 3-, 5-year OS after LR = 82.6%, 50.2%, 33.0%	​
Ito et al. [91]	Single-center retrospective Single center (UCLA) 1985–2019	LT for iCCA/pCCA	19 pCCA, 30 iCCA	Multimodal NAT is associated with improved survival in LT for both iCCA and hCCA regardless of tumor size	5-year OS after LT (2008–2019) for pCCA = 88% with NCT, 9% without NCT, for iCCA = 100% with NCT, 41% without NCT.	Survival: Neoadjuvant treatment, era of treatment, multifocal tumors, grading
McMillan et al. [93]	Retrospective cohort Single center (MD Anderson) 2010–2021	MD Anderson LT protocol for locally advanced iCCA	18	LT could be a treatment for highly selected patients with locally advanced, unresectable iCCA, after NCT with disease stability for at least 6 months	1-, 3-, and 5-year OS = 100%, 71%, and 57%. 1-, 3-year RFS = 70% and 52%	​
Kim et al. [103]	Retrospective Multicenter (national cancer database) 2004–2016	LT and LR for incidental iCCA	66	LT is effective in select patients with localized iCCA. No difference in OS and RFS between LT and LR.	5-year OS after LT = 36.1%, after LR = 32.7%, after CT alone = 5.3%	​
Lee et al. [104]	Multicenter (database) 2004–2018	Disparities in treatment for early iCCA	62 LT	LT had a trend toward improved OS compared to LR.	1-, 3-, and 5-year OS after LT = 88.9%, 72.9% and 67.9% (95% CI: 55.8%–82.5%)	​
Huang et al. [106]	Retrospective, Multicenter, SEER database analysis 2000–2019	LT for iCCA vs. LR for iCCA vs. LT for HCC	113 LT; 2425 LR; 5048 LT HCC	Patients with ICC after LT had a better prognosis than those after LR but inferior to HCC after LT	5-y OS: LT iCCA = 52.8%, LR iCCA = 29.9%; LT iCCA = 61.7% in patients with local advanced ICC after NCT.	​
Howell et al. [105]	Multicenter (national cancer database; UNOS STAR) 2010–2018	LT and LR for iCCA	153 LT	LR remains the standard of care for patients with resectable disease. Highly selected patients with unresectable iCCA may achieve favorable outcomes after LT.	5-year OS after LT = 59.8%, after LR = 39.9%. mOS after LT = 105.7 months	Survival: older age, other race (vs. White), stage II and III disease (vs. stage I), and presence of comorbidities, receiving surgery at an academic center, more recent year of diagnosis

## Looking to the future: patient selection through the lens of biological agressiveness

**FIGURE 4:**
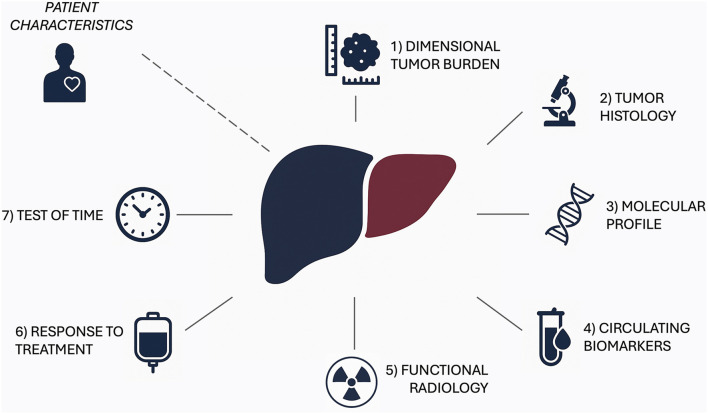
The seven pillars of biological aggressiveness.

The most challenging task in transplant oncology [17] is not to “extend criteria” for transplantation but, on the contrary, to improve their predictive capabilities by moving beyond static morphological parameters towards dynamic, biology-driven multiparametric decision-making (Figure 4). Understanding tumor biology remains a significant challenge due to its inherent complexity and heterogeneity, complicating the identification of consistent prognostic patterns. Both tumor-related and patient-specific factors contribute to posttransplant clinical outcomes. In synthesis, seven key pillars can be identified as indicators of biological aggressiveness: 1) tumor burden, 2) tumor histology, 3) molecular profile 4) circulating biomarkers 5) functional radiology 6) response to treatment 7) test of time. When corroborated by sufficient evidence, data related to these pillars will be integrated with prognostically relevant patient variables impacting on post-transplant survival to draw a personalized, multidisciplinary driven, pattern of transplantability aimed at guiding the decision making process. AI will be critical in such an evolution [109, 110].

### Histology and molecular profiling

Tumor burden cutoffs and patterns are already included in most LT protocols for patient selection, both in pCCA [6, 38] and iCCA [84].

Even though not yet integrated in patient selection processes, molecular biology might play a crucial role. In particular, iCCA exhibits marked heterogeneity, with significant diagnostic, prognostic, and therapeutic implications. Small-duct and large-duct subtypes, differ in morphology, cellular origin, clinical behavior, and molecular characteristics. Small-duct subtype generally confers better prognosis and is often associated with targetable genetic alterations (IDH1/IDH2, FGFR2). In contrast, large-duct iCCA more commonly harbors classical adenocarcinoma genetic alterations, such as in KRAS and TP53 [11]. Next-generation sequencing enables detection of actionable mutations, and is gradually transforming oncologic care [111]. A large meta-analysis of 1,481 resected iCCA cases demonstrated that patients with tumors harboring KRAS, TP53, and/or SMAD4 mutations had significantly worse OS and RFS compared to those with FGFR2 fusions, IDH mutations, BAP1 mutations, or no major genetic abnormalities [112]. Additional mutations in RB1, ERBB2, and BAP1 are also frequently observed in iCCA, and up to 70% of patients harbor potentially targetable genetic alterations [113]. Pemigatinib and infigratinib, showed progression-free survival (PFS) of 6.9 and 5.8 months, respectively, in patients with FGFR2 fusions [114–116]. Ivosidenib (IDH1 inhibitor) improved both PFS and OS in the ClarIDHy trial and was approved as palliative treatment [117, 118].

### Circulating biomarkers and liquid biopsy

Ca19-9 is elevated in 60%–80% [19, 119] of cases, and its diagnostic accuracy is limited by false positives in biliary obstruction and cholangitis. High preoperative and postoperative values of CA19-9, particularly when associated with increased carcinoembryonic antigen (CEA), are linked with advanced disease and worse OS and RFS [120–122]. On the contrary, a >50% reduction in CA19-9 after systemic therapy is strongly associated with radiologic response [120] and improved survival, and could serve as therapeutic objective.

Liquid biopsy is a non-invasive technique [2, 123] enabling detection of circulating tumor-derived material, including circulating tumor cells, ctDNA, ctRNA, microRNAs, and extracellular vesicles. ctDNA has emerged as a key biomarker for genomic profiling and tumor burden assessment [123–125], showing high concordance with tissue mutation profiles [126–128], with variant allele frequencies correlating with tumor load and supporting its role as a dynamic indicator of disease status [124, 129, 130]. ctDNA is detectable across all disease stages and carries prognostic value, with ctDNA-positive patients showing poorer progression-free survival both pre- and postoperatively. During surveillance, ctDNA detection is associated with significantly worse relapse-free survival and identifies recurrence in 93.8% of cases, with a mean lead time of 3.7 months over imaging [131].

Bile-derived cfDNA appears particularly promising due to direct tumor contact, detecting driver mutations in 54% of cases compared with 17% in plasma [132, 133].

However, limitations include lower sensitivity for gene fusions compared with tissue RNA-based assays, variability in ctDNA shedding depending on tumor burden and site, lack of standardization in extraction methods, platforms and timing, need for prospective interventional validation, and cost and reimbursement issues [123–125]. ctDNA remains complementary rather than a replacement for tissue testing, increasing actionable variant detection by 14.3% when used concurrently, and is particularly valuable when tissue is insufficient, unavailable, or when rapid or serial assessment is required [126, 128, 134].

### Functional radiology

Radiomics-based machine learning shows excellent diagnostic accuracy [135, 136] in CCA, particularly when integrated into clinical–radiomic models [137], often achieving performance comparable to postoperative pathology. Its main strength lies in improving diagnosis and preoperative prediction of microvascular invasion [138–140], gene mutations, perineural invasion [141], and lymph node metastasis [142, 143]. This stratification may guide surgical decision-making and enable prediction of early recurrence [144, 145] and survival [146]. Emerging deep learning models enable multimodal integration of radiology, pathology, and molecular data, but remain limited by data heterogeneity, poor interpretability, lack of standardization, and the need for prospective multicenter validation. Despite expert-level performance, clinical translation is hindered by regulatory constraints, cost sustainability, algorithmic bias, and insufficient validation [137, 147, 148].

Functional and metabolic assessment could provide useful insights as shown in CRLM transplantation setting [149]. PET is not recommended [48] for tumor diagnosis due to limited accuracy, but shows good performance in detecting lymph node and distant metastases [150–154]. SUVmax is an independent prognostic factor for disease-free and overall survival [155–159]. While 18F-FDG PET/CT is established for detecting metastatic disease and recurrence with high specificity, PET/MRI provides superior staging accuracy, particularly for T and N staging [160–162]. A new tracer 68Ga-FAPI PET/CT, targeting cancer-associated fibroblasts in the tumor microenvironment, shows high positive predictive value, high detection rates and better outcomes compared to 18F-FDG in terms of detection of primary tumors, lymph nodes, and distant metastases [163–168].

### Response to therapy and test of time

Integrating the patient’s clinical trajectory into the selection algorithm provides a longitudinal perspective on disease behavior [169]. The “test of time” itself reflects an indolent tumor biology, characterized by disease confinement within the liver and the absence of systemic or circulating tumor spread. Similarly, response to therapy serves as a surrogate marker of favorable tumor biology and informs postoperative management, as patients who respond to treatment before transplantation are more likely to maintain therapeutic sensitivity thereafter. For these reasons, as in the setting of CRLM [170], these principles have been incorporated into most modern neoadjuvant protocols, emphasizing refined patient selection over procedural acceleration [39, 93].

## Conclusion

Liver transplantation for cholangiocarcinoma has evolved from contraindication to a viable option in highly selected patients, with outcomes comparable to other oncologic indications when strict criteria are applied. Interpretation of the available evidence is limited by the predominance of retrospective studies, small and highly selected cohorts, and significant heterogeneity in neoadjuvant strategies and selection criteria across centers. In addition, most data derive from highly specialized institutions reporting limited case volumes over prolonged periods, and prospective validation of emerging biomarkers remains scarce, further restricting the generalizability of current findings. Evidence indicates that prognosis is driven primarily by tumor biology, response to therapy, and disease stability rather than anatomical factors alone. While standardized protocols define current practice in pCCA and selected indications are emerging in iCCA, recurrence risk, dropout rates, and organ scarcity remain major limitations.

The field is shifting toward a multiparametric, biology-driven model of transplantability integrating clinical, radiologic, and molecular data, although prospective validation is still required. We envision a future in which patient selection is guided by an integrated assessment of validated morpho-biologic prognostic parameters together with general preoperative predictors emerging from the pretransplant evaluation. The development of such complex “transplantability patterns”, rather than simplistic in/out criteria, will be crucial and will most likely be co-piloted by AI-based decision support. Overall, future progress will depend on refining selection, validating ongoing trials, and balancing oncologic benefit with equitable graft allocation. At the same time, the potential for an increase of oncologic indications for LT enhances competition for the limited organ supply [171]. To address this, further efforts should be made on expanding the donor pool through extended-criteria donors, LDLT and techniques of liver splitting and graft mitigation/manipulation [71, 72, 172–174].

## References

[1] Allkushi E Wehrle CJ Kim JK Khalil M Kwon DCH Fujiki M Expanding indications in transplant oncology. Cancers (Basel) (2025) 17(5):773. 10.3390/cancers17050773 40075625 PMC11898796

[2] Karageorgos FF Karakasi KE Kofinas A Antoniadis N Katsanos G Tsoulfas G . Evolving transplant oncology: evolving criteria for better decision-making. Diagnostics (2025) 15(7):820. 10.3390/diagnostics15070820 40218170 PMC11988714

[3] Krendl FJ Bellotti R Sapisochin G Schaefer B Tilg H Scheidl S Transplant oncology –Current indications and strategies to advance the field. JHEP Rep. (2023). 6(2):100965. 10.1016/j.jhepr.2023.100965 38304238 PMC10832300

[4] European Association for the Study of the Liver. EASL-ILCA clinical practice guidelines on he management of intrahepatic cholangiocarcinoma. J Hepatol. (2023) 79(1):181–208. 10.1016/j.jhep.2023.03.010 37084797

[5] Vogel A Bridgewater J Edeline J Kelley RK Klümpen HJ Malka D Biliary tract cancer: ESMO clinical practice guideline for diagnosis, treatment and follow-up ☆. Ann Oncol (2023) 34(2):127–40. 10.1016/j.annonc.2022.10.506 36372281

[6] Pfister M Ratti F Gores GJ Lesurtel M Chiche L Ebata T Recommendations on perihilar cholangiocarcinoma. The milan jury-based consensus. Ann Surg (2025). 10.1097/SLA.0000000000006773 40478744

[7] Sapisochin G Hibi T Toso C Man K Berenguer M Heimbach J Transplant oncology in primary and metastatic liver tumors: principles, evidence, and opportunities. Ann Surg (2021) 273(3):483–93. 10.1097/SLA.0000000000004071 33065633

[8] Hibi T Rela M Eason JD Line PD Fung J Sakamoto S Liver transplantation for colorectal and neuroendocrine liver metastases and hepatoblastoma. Working group report from the ILTS transplant oncology consensus conference. Transplantation (2020) 104(6):1131–5. 10.1097/TP.0000000000003118 32217939

[9] Adam R Piedvache C Chiche L Adam JP Salamé E Bucur P Liver transplantation plus chemotherapy versus chemotherapy alone in patients with permanently unresectable colorectal liver metastases (TransMet): results from a multicentre, open-label, prospective, randomised controlled trial. The Lancet (2024) 404(10458):1107–18. 10.1016/S0140-6736(24)01595-2 39306468

[10] Cillo U Gringeri E D’Amico FE Lanari J Furlanetto A Vitale A . Hepatocellular carcinoma: revising the surgical approach in light of the concept of multiparametric therapeutic hierarchy. Dig Liver Dis (2025) 57(4):809–18. 10.1016/j.dld.2024.12.003 39828438

[11] Banales JM Marin JJG Lamarca A Rodrigues PM Khan SA Roberts LR Cholangiocarcinoma 2020: the next horizon in mechanisms and management. Nat Rev Gastroenterol Hepatol (2020) 17(9):557–88. 10.1038/s41575-020-0310-z 32606456 PMC7447603

[12] Vithayathil M Khan SA . Current epidemiology of cholangiocarcinoma in Western countries. J Hepatol (2022) 77(6):1690–8. 10.1016/j.jhep.2022.07.022 35977611

[13] Rimassa L Lamarca A O’Kane GM Edeline J McNamara MG Vogel A New systemic treatment paradigms in advanced biliary tract cancer and variations in patient access across Europe. The Lancet Reg Health - Europe (2025) 50:101170. 10.1016/j.lanepe.2024.101170 PMC11910789 40093395

[14] Malik AK Davidson BR Manas DM . Surgical management, including the role of transplantation, for intrahepatic and peri-hilar cholangiocarcinoma. Eur J Surg Oncol (2025) 51(2):108248. 10.1016/j.ejso.2024.108248 38467524

[15] Esmail A Badheeb M Alnahar B Almiqlash B Sakr Y Khasawneh B Cholangiocarcinoma: the current status of surgical options including liver transplantation. Cancers (Basel) (2024) 16(11):1946. 10.3390/cancers16111946 38893067 PMC11171350

[16] Kodali S Kulik L D’Allessio A De Martin E Hakeem AR Lewinska M The 2024 ILTS-ILCA consensus recommendations for liver transplantation for HCC and intrahepatic cholangiocarcinoma. Liver Transplant (2025) 31(6):815–31. 10.1097/LVT.0000000000000589 40014003

[17] Connor AA Kodali S Abdelrahim M Javle MM Brombosz EW Ghobrial RM . Intrahepatic cholangiocarcinoma: the role of liver transplantation, adjunctive treatments, and prognostic biomarkers. Front Oncol (2022) 12:996710. 10.3389/fonc.2022.996710 36479082 PMC9719919

[18] Cillo U Fondevila C Donadon M Gringeri E Mocchegiani F Schlitt HJ Surgery for cholangiocarcinoma. Liver Int (2019) 39(Suppl. 1):143–55. 10.1111/LIV.14089 30843343 PMC6563077

[19] Izquierdo-Sanchez L Lamarca A La Casta A Buettner S Utpatel K Klümpen HJ Cholangiocarcinoma landscape in Europe: diagnostic, prognostic and therapeutic insights from the ENSCCA registry. J Hepatol (2022) 76(5):1109–21. 10.1016/j.jhep.2021.12.010 35167909

[20] Alaimo L Endo Y Catalano G Ruzzenente A Aldrighetti L Weiss M Benchmarks in liver resection for intrahepatic cholangiocarcinoma. Ann Surg Oncol (2024) 31(5):3043–52. 10.1245/s10434-023-14880-8 38214817 PMC10997542

[21] Mueller M Breuer E Mizuno T Bartsch F Ratti F Benzing C Perihilar cholangiocarcinoma – novel benchmark values for surgical and oncological outcomes from 24 expert centers. Ann Surg (2021) 274(5):780–8. 10.1097/SLA.0000000000005103 34334638

[22] Cillo U Furlanetto A Nieddu E Polacco M Boetto R Bassi D Ante situm liver surgery using machine perfusion liver preservation: pilot human experience. Br J Surg (2021) 108:e235–e236. 10.1093/bjs/znab095 33824957

[23] Cillo U D’Amico FE Furlanetto A Perin L Gringeri E . Robotic hepatectomy and biliary reconstruction for perihilar cholangiocarcinoma: a pioneer western case series. Updates Surg Springer Sci Business Media Deutschland GmbH (2021) 73(3):999–1006. 10.1007/s13304-021-01041-3 PMC8184707 33861401

[24] Weigt J Malfertheiner P . Cisplatin plus gemcitabine versus gemcitabine for biliary tract cancer. Expert Rev Gastroenterol Hepatol (2010) 4(4):395–7. 10.1586/egh.10.45 20678012

[25] Oh DY Ruth He A Qin S Chen LT Okusaka T Vogel A Durvalumab plus gemcitabine and cisplatin in advanced biliary tract cancer. NEJM Evid (2022) 1(8):EVIDoa2200015. 10.1056/evidoa2200015 38319896

[26] Li Z Aliseda D Jones O Rajendran L Magyar C Grant R Recent advances in systemic therapy for advanced biliary tract cancer: a systematic review and meta-analysis using reconstructed RCT survival data. JHEP Rep (2025) 7(3):101290. 10.1016/j.jhepr.2024.101290 39980751 PMC11840543

[27] Oh DY He AR Bouattour M Okusaka T Qin S Chen LT Durvalumab or placebo plus gemcitabine and cisplatin in participants with advanced biliary tract cancer (TOPAZ-1): updated overall survival from a randomised phase 3 study. Lancet Gastroenterol Hepatol (2024) 9(8):694–704. 10.1016/S2468-1253(24)00095-5 38823398

[28] Kelley RK Ueno M Yoo C Finn RS Furuse J Ren Z Pembrolizumab in combination with gemcitabine and cisplatin compared with gemcitabine and cisplatin alone for patients with advanced biliary tract cancer (KEYNOTE-966): a randomised, double-blind, placebo-controlled, phase 3 trial. The Lancet (2023) 401(10391):1853–65. 10.1016/S0140-6736(23)00727-4 37075781

[29] Wilbur HC Soares HP Azad NS . Neoadjuvant and adjuvant therapy for biliary tract cancer: advances and limitations. Hepatology (2024) 82:1287–302. 10.1097/HEP.0000000000000760 38266282

[30] Goetze TO Vogel A Pratschke J Behrend M Reim D Schnitzbauer AA Neoadjuvant chemotherapy with gemcitabine plus cisplatin followed by radical liver resection versus immediate radical liver resection alone followed adjuvant therapy in biliary tract cancer: final results from the phase III AIO/CALGP/ACO-GAIN-Trial. J Clin Oncol (2025) 43(16_Suppl. l):4008. 10.1200/JCO.2025.43.16_SUPPL.4008

[31] Ratti F Marino R Olthof PB Pratschke J Erdmann JI Neumann UP Predicting futility of upfront surgery in perihilar cholangiocarcinoma: machine learning analytics model to optimize treatment allocation. Hepatology (2024) 79(2):341–54. 10.1097/HEP.0000000000000554 37530544

[32] Bhat M Hathcock M Kremers WK Darwish Murad S Schmit G Martenson J Portal vein encasement predicts neoadjuvant therapy response in liver transplantation for perihilar cholangiocarcinoma protocol. Transpl Int (2015) 28(12):1383–91. 10.1111/tri.12640 26183487

[33] Meyer CG Penn I James L . Liver transplantation for cholangiocarcinoma: results in 207 patients. Transplantation (2000) 69(8):1633–7. 10.1097/00007890-200004270-00019 10836374

[34] Robles R Figueras J Turrión VS Margarit C Moya A Varo E Spanish experience in liver transplantation for hilar and peripheral cholangiocarcinoma. Ann Surg (2004) 239(2):265–71. 10.1097/01.sla.0000108702.45715.81 14745336 PMC1356221

[35] Seehofer D Thelen A Neumann UP Veltzke-Schlieker W Denecke T Kamphues C Extended bile duct resection and [corrected] liver and transplantation in patients with hilar cholangiocarcinoma: long-term results. Liver Transpl (2009) 15(11):1499–507. 10.1002/lt.21887 19877250

[36] Ghali P Marotta PJ Yoshida EM Bain VG Marleau D Peltekian K Liver transplantation for incidental cholangiocarcinoma: analysis of the Canadian experience. Liver Transpl (2005) 11(11):1412–6. 10.1002/lt.20512 16237695

[37] Heimbach JK Gores GJ Haddock MG Alberts SR Nyberg SL Ishitani MB Liver transplantation for unresectable perihilar cholangiocarcinoma. Semin Liver Dis (2004) 24(2):201–7. 10.1055/s-2004-828896 15192792

[38] Rosen CB Heimbach JK Gores GJ . Liver transplantation for cholangiocarcinoma. Transpl Int (2010) 23(7):692–7. 10.1111/j.1432-2277.2010.01108.x 20497401

[39] Gringeri E Furlanetto A Billato I Cescon M De Carlis L Mazzaferro V The Italian experience on liver transplantation for unresectable peri-hilar cholangiocarcinoma: a national survey and future perspectives. Updates Surg (2024) 76(7):2505–13. 10.1007/s13304-024-01889-1 39210194

[40] Heimbach JK Sanchez W Rosen CB Gores GJ . Trans-peritoneal fine needle aspiration biopsy of hilar cholangiocarcinoma is associated with disease dissemination. Hpb (2011) 13(5):356–60. 10.1111/j.1477-2574.2011.00298.x 21492336 PMC3093648

[41] Darwish MS Kim WR Harnois DM Douglas DD Burton J Kulik LM Efficacy of neoadjuvant chemoradiation, followed by liver transplantation, for perihilar cholangiocarcinoma at 12 US centers. Gastroenterology (2012) 143(1):88–98. 10.1053/j.gastro.2012.04.008 22504095 PMC3846443

[42] Tan EK Taner T Heimbach JK Gores GJ Rosen CB . Liver transplantation for peri-hilar cholangiocarcinoma. J Gastrointest Surg (2020) 24(11):2679–85. 10.1007/s11605-020-04721-4 32671802

[43] Cambridge WA Fairfield C Powell JJ Harrison EM Søreide K Wigmore SJ Meta-analysis and meta-regression of survival after liver transplantation for unresectable perihilar cholangiocarcinoma. Ann Surg (2021) 273(2):240–50. 10.1097/SLA.0000000000003801 32097164

[44] Mantel HTJ Westerkamp AC Adam R Bennet WF Seehofer D Settmacher U Strict selection alone of patients undergoing liver transplantation for hilar cholangiocarcinoma is associated with improved survival. PLoS One (2016) 11(6):e0156127. 10.1371/journal.pone.0156127 27276221 PMC4898828

[45] Hoogwater FJH Kuipers H De Meijer VE Maulat C Muscari F Polak WG Role of neoadjuvant chemoradiotherapy in liver transplantation for unresectable perihilar cholangiocarcinoma: Multicentre, retrospective cohort study. BJS Open (2023) 7(2):1–8. 10.1093/bjsopen/zrad025 37032423 PMC10083139

[46] Dong Y Li Z Podrascanin V Eaton JE Ilyas SI Gores GJ Liver resection with and without vascular resection versus transplantation for *de novo* perihilar cholangiocarcinoma. Hepatology (2025) 83:1128–42. 10.1097/HEP.0000000000001449 40622853 PMC13089817

[47] Mantel HTJ Rosen CB Heimbach JK Nyberg SL Ishitani MB Andrews JC Vascular complications after orthotopic liver transplantation after neoadjuvant therapy for hilar cholangiocarcinoma. Liver Transpl (2007) 13(10):1372–81. 10.1002/lt.21107 17427173

[48] Bowlus CL Arrivé L Bergquist A Deneau M Forman L Ilyas SI AASLD practice guidance on primary sclerosing cholangitis and cholangiocarcinoma. Hepatology (2023) 77(2):659–702. 10.1002/HEP.32771 36083140

[49] Jansson H Olthof PB Bergquist A Ligthart MAP Nadalin S Troisi RI Outcome after resection for perihilar cholangiocarcinoma in patients with primary sclerosing cholangitis: an international multicentre study. HPB (2021) 23(11):1751–8. 10.1016/j.hpb.2021.04.011 33975797 PMC8720371

[50] Are C Dhir M Ravipati L . History of pancreaticoduodenectomy: early misconceptions, initial milestones and the pioneers. HPB (2011) 13(6):377–84. 10.1111/j.1477-2574.2011.00305.x 21609369 PMC3103093

[51] Miyagawa S Makuuchi M Kawasaki S Hayashi K Harada H Kitamura H Outcome of major hepatectomy with pancreatoduodenectomy for advanced biliary malignancies. World J Surg (1996) 20(1):77–80. 10.1007/s002689900014 8588418

[52] Ebata T Yokoyama Y Igami T Sugawara G Takahashi Y Nimura Y Hepatopancreatoduodenectomy for cholangiocarcinoma: a single-center review of 85 consecutive patients. Ann Surg (2012) 256(2):297–305. 10.1097/SLA.0b013e31826029ca 22750757

[53] Ishii T Seo S Ito T Ogiso S Fukumitsu K Masui T Liver transection-first approach in hepatopancreatoduodenectomy for hilar cholangiocarcinoma: a safe and secure technique for the early assessment of curable resection and vascular reconstruction. Ann Surg Oncol (2021) 28(6):2988–9. 10.1245/s10434-020-09303-x 33169301

[54] Chiba N Abe Y Yokozuka K Hikita K Kobayashi T Sano T Surgical technique of pancreatic parenchyma transection-delayed approach (PPTDA) in hepatopancreatoduodenectomy for hilar cholangiocarcinoma. J Gastrointest Surg (2019) 23(3):613–6. 10.1007/s11605-018-3923-6 30187328

[55] Yoshimi Y Noji T Okamura K Tanaka K Matsui A Nakanishi Y The Short- and long-term surgical results of consecutive hepatopancreaticoduodenectomy for wide-spread biliary malignancy. Ann Surg Oncol (2023) 31(1):90–6. 10.1245/s10434-023-14406-2 37899414

[56] Sugiura T Ohgi K Ashida R Yamada M Kato Y Otsuka S Hepatopancreatoduodenectomy for extrahepatic cholangiocarcinoma: a series of 100 consecutive cases from an expert center in Japan. Ann Surg Oncol (2025) 32(9):6531–40. 10.1245/s10434-025-17515-2 40399599

[57] Shimizu A Motoyama H Kubota K Notake T Fukushima K Ikehara T Safety and oncological benefit of hepatopancreatoduodenectomy for advanced extrahepatic cholangiocarcinoma with horizontal tumor spread: shinshu university experience. Ann Surg Oncol (2021) 28(4):2012–25. 10.1245/s10434-020-09209-8 33044629

[58] Endo I Hirahara N Miyata H Yamamoto H Matsuyama R Kumamoto T Mortality, morbidity, and failure to rescue in hepatopancreatoduodenectomy: an analysis of patients registered in the national clinical database in Japan. J Hepatobiliary Pancreat Sci (2021) 28(4):305–16. 10.1002/jhbp.918 33609319

[59] Kiritani S Kawaguchi Y Nishioka Y Mihara Y Ichida A Takamoto T Long-term outcomes of hepatopancreatoduodenectomy for perihilar cholangiocarcinoma: a comparative study with conventional hepatectomy. Eur J Surg Oncol (2025) 51(5):109633. 10.1016/j.ejso.2025.109633 39892087

[60] Yasukawa K Shimizu A Kubota K Notake T Hosoda K Sakai H Reassessing the role of hepatopancreatoduodenectomy in advanced biliary tract cancer: a systematic review and single-arm meta-analysis of modern case series. Ann Surg Oncol (2026) 33(2):1515–25. 10.1245/s10434-025-18665-z 41212458

[61] Aoki T Sakamoto Y Kohno Y Akamatsu N Kaneko J Sugawara Y Hepatopancreaticoduodenectomy for biliary cancer: strategies for near-zero operative mortality and acceptable long-term outcome. Ann Surg (2018) 267(2):332–7. 10.1097/SLA.0000000000002059 27811506

[62] Welch JC Gleeson EM Karachristos A Pitt HA . Hepatopancreatoduodenectomy in North America: are the outcomes acceptable? HPB (2020) 22(3):360–7. 10.1016/j.hpb.2019.08.010 31519357

[63] D’Souza MA Valdimarsson VT Campagnaro T Cauchy F Chatzizacharias NA D'Hondt M Hepatopancreatoduodenectomy –a controversial treatment for bile duct and gallbladder cancer from a European perspective. HPB (2020) 22(9):1339–48. 10.1016/j.hpb.2019.12.008 31899044

[64] Stauffer JA Steers JL Bonatti H Dougherty MK Aranda-Michel J Dickson RC Liver transplantation and pancreatic resection: a single-center experience and a review of the literature. Liver Transpl (2009) 15(12):1728–37. 10.1002/lt.21932 19938125

[65] Soejima Y Ueda S Sanefuji K Kayashima H Yoshizumi T Ikegami T Sequential pancreaticoduodenectomy after living donor liver transplantation for cholagiocacinoma. Am J Transplant (2008) 8(10):2158–62. 10.1111/j.1600-6143.2008.02346.x 18727703

[66] Varotti G Gondolesi GE Roayaie S Suriawinata A Soltys K Fishbein TM Combined adult-to-adult living donor right lobe liver transplantation and pancreatoduodenectomy for distal bile duct adenocarcinoma in a patient with primary sclerosing cholangitis. J Am Coll Surg (2003) 197(5):765–9. 10.1016/j.jamcollsurg.2003.06.001 14585411

[67] Gringeri E Bassi D D’Amico FE Boetto R Polacco M Lodo E Neoadjuvant therapy protocol and liver transplantation in combination with pancreatoduodenectomy for the treatment of hilar cholangiocarcinoma occurring in a case of primary sclerosing cholangitis: case report with a more than 8-year disease-free surviva. Transpl Proc (2011) 43(4):1187–9. 10.1016/j.transproceed.2011.01.140 21620084

[68] Wu Y Johlin FC Rayhill SC Jensen CS Xie J Cohen MB Long-term, tumor-free survival after radiotherapy combining hepatectomy-whipple *en bloc* and orthotopic liver transplantation for early-stage hilar cholangiocarcinoma. Liver Transplant (2008) 14(3):279–86. 10.1002/lt.21287 18306329

[69] Moon DB Lee SG Kim KH . Total hepatectomy, pancreatoduodenectomy, and living donor liver transplantation using innovative vascular reconstruction for unresectable cholangiocarcinoma. Transpl Int (2015) 28(1):123–6. 10.1111/tri.12401 25041446

[70] Ito T Taura K Fukumitsu K Okumura S Ogiso S Anazawa T Safety and efficacy of living donor liver transplantation for unresectable perihilar cholangiocarcinoma: a single center prospective study. J Hepatobiliary Pancreat Sci (2025) 32(4):276–86. 10.1002/jhbp.12121 39996522 PMC12038382

[71] Cillo U Furlanetto A Gringeri E Bertacco A Marchini A Rosso E Advocating for a “ shift - to - left ” in transplant oncology: left grafts, RAPID and dual graft. Updates Surg (2024) 77(0123456789):1889–902. 10.1007/s13304-024-01919-y 39120859

[72] Gringeri E Furlanetto A Polacco M Perin L Nieddu E Rosso E Exploring auxiliary liver transplantation in the era of transplant oncology-A proposal for a new liver splitting program (ALERT-50). Liver Transpl (2025) 31(9):1176–82. 10.1097/LVT.0000000000000574 39835850

[73] Tan EK Rosen CB Heimbach JK Gores GJ Zamora-Valdes D Taner T . Living donor liver transplantation for perihilar cholangiocarcinoma: outcomes and complications. J Am Coll Surg (2020) 231(1):98–110. 10.1016/j.jamcollsurg.2019.12.037 32035181

[74] Moris D Kostakis ID Machairas N Prodromidou A Tsilimigras DI Ravindra KV Comparison between liver transplantation and resection for hilar cholangiocarcinoma: a systematic review and meta-analysis. PLoS One (2019) 14(7):1–13. 10.1371/journal.pone.0220527 31365594 PMC6668826

[75] Breuer E Mueller M Doyle MB Yang L Darwish Murad S Anwar IJ Liver transplantation as a new standard of care in patients with perihilar cholangiocarcinoma? Results from an international benchmark study. Ann Surg (2022) 276(5):846–53. 10.1097/SLA.0000000000005641 35894433 PMC9983747

[76] Muller X Marcon F Sapisochin G Marquez M Dondero F Rayar M Defining benchmarks in liver transplantation: a multicenter outcome analysis determining best achievable results. Ann Surg (2018) 267(3):419–25. 10.1097/SLA.0000000000002477 28885508

[77] Gringeri E Furlanetto A Nieddu E Romagnoli R Gruttadauria S Ghinolfi D From clinical heterogeneity to national standardization in liver transplantation for perihilar cholangiocarcinoma: the LITALHICA protocol. Transplantation (2026). in press. 10.1097/TP.0000000000005762 42418260

[78] Heimbach JK Gores GJ Haddock MG Alberts SR Pedersen R Kremers W Predictors of disease recurrence following neoadjuvant chemoradiotherapy and liver transplantation for unresectable perihilar cholangiocarcinoma. Transplantation (2006) 82(12):1703–7. 10.1097/01.tp.0000253551.43583.d1 17198263

[79] Murad SD Kim WR Therneau T Gores GJ Rosen CB Martenson JA Predictors of pretransplant dropout and posttransplant recurrence in patients with perihilar cholangiocarcinoma. Hepatology (2012) 56(3):972–81. 10.1002/hep.25629 22290335 PMC3830980

[80] Croome KP Rosen CB Heimbach JK Nagorney DM . Is liver transplantation appropriate for patients with potentially resectable *de novo* hilar cholangiocarcinoma? J Am Coll Surg (2015) 221(1):130–9. 10.1016/j.jamcollsurg.2015.01.064 25872685

[81] Ethun CG Lopez-Aguiar AG Anderson DJ Adams AB Fields RC Doyle MB Transplantation versus resection for hilar cholangiocarcinoma: an argument for shifting treatment paradigms for resectable disease. Ann Surg (2018) 267(5):797–805. 10.1097/SLA.0000000000002574 29064885 PMC6002861

[82] Zaborowski A Heneghan HM Fiore B Stafford A Gallagher T Geoghegan J Neoadjuvant chemoradiotherapy and liver transplantation for unresectable hilar cholangiocarcinoma: the Irish experience of the Mayo protocol. Transplantation (2020) 104(10):2097–104. 10.1097/TP.0000000000003114 31972704

[83] Sapisochin G De Lope CR Gastaca M de Urbina JO López-Andujar R Palacios F Intrahepatic cholangiocarcinoma or mixed hepatocellular-cholangiocarcinoma in patients undergoing liver transplantation: a spanish matched cohort multicenter study. Ann Surg (2014) 259(5):944–52. 10.1097/SLA.0000000000000494 24441817

[84] Sapisochin G Facciuto M Rubbia-Brandt L Marti J Mehta N Yao FY Liver transplantation for “very early” intrahepatic cholangiocarcinoma: international retrospective study supporting a prospective assessment. Hepatology (2016) 64(4):1178–88. 10.1002/hep.28744 27481548

[85] Ziogas IA Giannis D Economopoulos KP Hayat MH Montenovo MI Matsuoka LK Liver transplantation for intrahepatic cholangiocarcinoma: a meta-analysis and meta-regression of survival rates. Transplantation (2021) 105(10):2263–71. 10.1097/TP.0000000000003539 33196623

[86] Tham EKJ Lim RY Koh B Tan DJH Ng CH Law M Prevalence of chronic liver disease in cholangiocarcinoma: a meta-analysis. Clin Gastroenterol Hepatol (2025) 23(10):1710–8. 10.1016/J.CGH.2024.09.028 39461458

[87] Thakral N Gonzalez T Nano O Shin SH Samuels S Hussein A . Cirrhosis in intrahepatic cholangiocarcinoma: prognostic importance and impact on survival. BMC Gastroenterol (2023) 23(1):151. 10.1186/S12876-023-02710-W 37179301 PMC10183123

[88] De Martin E Rayar M Golse N Dupeux M Gelli M Gnemmi V Analysis of liver resection *versus* liver transplantation on outcome of small intrahepatic cholangiocarcinoma and combined hepatocellular-cholangiocarcinoma in the setting of cirrhosis. Liver Transplant (2020) 26(6):785–98. 10.1002/lt.25737 32090444

[89] Hong JC Jones CM Duffy JP Petrowsky H Farmer DG French S Comparative analysis of resection and liver transplantation for intrahepatic and hilar cholangiocarcinoma: a 24-year experience in a single center. Arch Surg (2011) 146(6):683–9. 10.1001/ARCHSURG.2011.116 21690444

[90] Hong JC Petrowsky H Kaldas FM Farmer DG Durazo FA Finn RS Predictive index for tumor recurrence after liver transplantation for locally advanced intrahepatic and hilar cholangiocarcinoma. J Am Coll Surg (2011) 212(4):514–20. 10.1016/j.jamcollsurg.2010.12.005 21463781

[91] Ito T Butler JR Noguchi D Ha M Aziz A Agopian VG A 3-Decade, single-center experience of liver transplantation for cholangiocarcinoma: impact of era, tumor size, location, and neoadjuvant therapy. Liver Transpl (2022) 28(3):386–96. 10.1002/LT.26285 34482610

[92] Lunsford KE Javle M Heyne K Shroff RT Abdel-Wahab R Gupta N Liver transplantation for locally advanced intrahepatic cholangiocarcinoma treated with neoadjuvant therapy: a prospective case-series. Lancet Gastroenterol Hepatol (2018) 3(5):337–48. 10.1016/S2468-1253(18)30045-1 29548617

[93] McMillan RR Javle M Kodali S Saharia A Mobley C Heyne K Survival following liver transplantation for locally advanced, unresectable intrahepatic cholangiocarcinoma. Am J Transplant (2022) 22(3):823–32. 10.1111/ajt.16906 34856069

[94] Kodali S Connor AA Thabet S Brombosz EW Ghobrial RM . Liver transplantation as an alternative for the treatment of intrahepatic cholangiocarcinoma: past, present, and future directions. Hepatobiliary Pancreat Dis Int Elsevier (Singapore) Pte Ltd. (2024) 23(2):129–38. 10.1016/j.hbpd.2023.07.007 37517983

[95] Rauchfuß F Ali-Deeb A Rohland O Dondorf F Ardelt M Settmacher U . Living donor liver transplantation for intrahepatic cholangiocarcinoma. Curr Oncol (2022) 29(3):1932–8. 10.3390/curroncol29030157 35323357 PMC8947647

[96] Hara T Eguchi S Yoshizumi T Akamatsu N Kaido T Hamada T Incidental intrahepatic cholangiocarcinoma in patients undergoing liver transplantation: a multi-center study in Japan. J Hepatobiliary Pancreat Sci (2021) 28(4):346–52. 10.1002/JHBP.896 33464720

[97] Hafeez Bhatti AB Tahir R Qureshi NR Mamoon N Khan NY Zia HH . Living donor liver transplantation for intra hepatic cholangiocarcinoma. Ann Med Surg (2020) 57(June):82–4. 10.1016/j.amsu.2020.07.028 32728435 PMC7381427

[98] Sierra L Barba R Ferrigno B Goyes D Diaz W Patwardhan VR Living-donor liver transplant and improved post-transplant survival in patients with primary sclerosing cholangitis. J Clin Med (2023) 12(8):2807. 10.3390/JCM12082807 37109144 PMC10145248

[99] Pichlmayl R Lamesch P Weimann A Tusch G Ringe B . Surgical treatment of cholangiocellular carcinoma. World J Surg (1995) 19(1):83–8. 10.1007/BF00316984 7740815

[100] Weimann A Varnholt H Schlitt HJ Lang H Flemming P Hustedt C Retrospective analysis of prognostic factors after liver resection and transplantation for cholangiocellular carcinoma. Br J Surg (2000) 87(9):1182–7. 10.1046/j.1365-2168.2000.01532.x 10971425

[101] Jung DH Hwang S Song GW Ahn CS Moon DB Kim KH Clinicopathological features and prognosis of intrahepatic cholangiocarcinoma after liver transplantation and resection. Ann Transpl (2017) 22:42–52. 10.12659/AOT.901504 28123170 PMC12577132

[102] Hue JJ Rocha FG Ammori JB Hardacre JM Rothermel LD Chavin KD A comparison of surgical resection and liver transplantation in the treatment of intrahepatic cholangiocarcinoma in the era of modern chemotherapy: an analysis of the national cancer database. J Surg Oncol (2021) 123(4):949–56. 10.1002/jso.26370 33400841

[103] Kim P Littau M Baker TB Abdelsattar Z Tonelli C Bunn C Intrahepatic cholangiocarcinoma: is there a role for liver transplantation? Surgery (United States) (2022) 171(3):741–6. 10.1016/j.surg.2021.09.034 34895770

[104] Lee YT Singal AG Lauzon M Agopian VG Luu M Noureddin M Disparities in curative treatments and outcomes for early stage intrahepatic cholangiocarcinoma in the United States. Cancer (2022) 128(20):3610–9. 10.1002/cncr.34436 35997126 PMC9530023

[105] Howell TC Rhodin KE Shaw B Bao J Kanu E Masoud S Contemporary trends and outcomes after liver transplantation and resection for intrahepatic cholangiocarcinoma. J Gastrointest Surg (2024) 28(5):738–45. 10.1016/j.gassur.2024.02.029 38704208

[106] Huang G Song W Zhang Y Yu J Lv Y Liu K . Liver transplantation for intrahepatic cholangiocarcinoma: a propensity score-matched analysis. Sci Rep (2023) 13(1):10630. 10.1038/s41598-023-37896-2 37391482 PMC10313647

[107] Yaqub S Busund S Smedman TM Syversveen T Khan A Solheim JM Liver transplantation for locally advanced non-resectable intrahepatic cholangiocarcinoma treated with neoadjuvant therapy: early results from the TESLA trial. Br J Surg (2025) 112(3):znaf054. 10.1093/bjs/znaf054 40099404 PMC11914714

[108] Maspero M Sposito C Bongini MA Cascella T Flores M Maccauro M Liver transplantation for intrahepatic cholangiocarcinoma after chemotherapy and radioembolization: an intention-to-treat study. Transpl Int (2024) 37:13641. 10.3389/ti.2024.13641 39544321 PMC11560448

[109] Deeb M Gangadhar A Rabindranath M Rao K Brudno M Sidhu A The emerging role of generative artificial intelligence in transplant medicine. Am J Transplant (2024) 24(10):1724–30. 10.1016/J.AJT.2024.06.009 38901561

[110] Loupy A Preka E Chen X Wang H He J Zhang K . Reshaping transplantation with AI, emerging technologies and xenotransplantation. Nat Med (2025) 31(7):2161–73. 10.1038/S41591-025-03801-9 40659768

[111] Young SE Sritharan R Sia D . Genomic alterations in intrahepatic cholangiocarcinoma. Hepatoma Res (2023) 9(34):34. 10.20517/2394-5079.2023.37

[112] Wang XY Zhu WW Wang Z Huang JB Wang SH Bai FM Driver mutations of intrahepatic cholangiocarcinoma shape clinically relevant genomic clusters with distinct molecular features and therapeutic vulnerabilities. Theranostics (2022) 27(1):260–76. 10.7150/THNO.63417 34987644 PMC8690927

[113] Sia D Losic B Moeini A Cabellos L Hao K Revill K Massive parallel sequencing uncovers actionable FGFR2-PPHLN1 fusion and ARAF mutations in intrahepatic cholangiocarcinoma. Nat Commun (2015) 6:6087. 10.1038/NCOMMS7087 25608663

[114] Javle M Lowery M Shroff RT Weiss KH Springfeld C Borad MJ Phase II study of BGJ398 in patients with FGFR-altered advanced cholangiocarcinoma. J Clin Oncol (2018) 36(3):276–82. 10.1200/JCO.2017.75.5009 29182496 PMC6075847

[115] Abou-Alfa GK Sahai V Hollebecque A Vaccaro G Melisi D Al-Rajabi R Pemigatinib for previously treated, locally advanced or metastatic cholangiocarcinoma: a multicentre, open-label, phase 2 study. Lancet Oncol (2020) 21(5):671–84. 10.1016/S1470-2045(20)30109-1 32203698 PMC8461541

[116] Javle M Roychowdhury S Kelley RK Sadeghi S Macarulla T Weiss KH Infigratinib (BGJ398) in previously treated patients with advanced or metastatic cholangiocarcinoma with FGFR2 fusions or rearrangements: mature results from a multicentre, open-label, single-arm, phase 2 study. Lancet Gastroenterol Hepatol (2021) 6(10):803–15. 10.1016/S2468-1253(21)00196-5 34358484

[117] Abou-Alfa GK Macarulla T Javle MM Kelley RK Lubner SJ Adeva J Ivosidenib in IDH1-mutant, chemotherapy-refractory cholangiocarcinoma (ClarIDHy): a multicentre, randomised, double-blind, placebo-controlled, phase 3 study. Lancet Oncol (2020) 21(6):796–807. 10.1016/S1470-2045(20)30157-1 32416072 PMC7523268

[118] Zhu AX Macarulla T Javle MM Kelley RK Lubner SJ Adeva J Final overall survival efficacy results of ivosidenib for patients with advanced cholangiocarcinoma with IDH1 mutation: the phase 3 randomized clinical ClarIDHy trial. JAMA Oncol (2021) 7(11):1669–77. 10.1001/JAMAONCOL.2021.3836 34554208 PMC8461552

[119] Liang B Zhong L He Q Wang S Pan Z Wang T Diagnostic accuracy of serum CA19-9 in patients with cholangiocarcinoma: a systematic review and meta-analysis. Med Sci Monit (2015) 21:3555–63. 10.12659/MSM.895040 26576628 PMC4655615

[120] Cai QY Yang P Yang XL Zhang XH Guo LP Lu XY The association of carbohydrate antigen 19-9 response with radiologic response and survival in intrahepatic cholangiocarcinoma: a prospective cohort study. Cancer (2023) 129(19):2999–3009. 10.1002/CNCR.34854 37449788

[121] Endo Y Kawashima J Woldesenbet S Akabane M Ruzzente A Aldrighetti L Characteristics of “Very Early” intrahepatic cholangiocarcinoma undergoing liver resection and its relationship with carbohydrate antigen 19-9. J Surg Oncol (2025) 132(5):924–34. 10.1002/JSO.70071 40842313 PMC12501924

[122] Okada K Kobayashi T Kuroda S Mashima H Hashimoto M Tahara H Prognostic impact of pre- and postoperative tumor markers in patients with intrahepatic cholangiocarcinoma. Surg Today (2024) 54(2):177–85. 10.1007/S00595-023-02715-8 37340141

[123] Kotsifa E Saffioti F Mavroeidis VK . Cholangiocarcinoma: the era of liquid biopsy. World J Gastroenterol (2025) 31(11):104170. 10.3748/wjg.v31.i11.104170 40124277 PMC11924015

[124] de Scordilli M Bortolot M Torresan S Noto C Rota S Di Nardo P Precision oncology in biliary tract cancer: the emerging role of liquid biopsy. ESMO Open (2025) 10(5):105079. 10.1016/j.esmoop.2025.105079 40311184 PMC12084404

[125] Wang Y Li Y Liang Z Zhang Y Li T Tian C Circulating tumor DNA in cholangiocarcinoma: current clinical applications and future perspectives. Front Cell Dev Biol (2025) 13:1616064. 10.3389/fcell.2025.1616064 40673275 PMC12263599

[126] Mahadevia H Majeed U Patel J Ahmed AK Elhariri A Albelal D Circulating tumor DNA and tissue testing for pancreatobiliary tumors. JAMA Netw Open (2025) 8(9):e2531373. 10.1001/jamanetworkopen.2025.31373 40938601 PMC12432637

[127] Ettrich TJ Schwerdel D Dolnik A Beuter F Blätte TJ Schmidt SA Genotyping of circulating tumor DNA in cholangiocarcinoma reveals diagnostic and prognostic information. Scientific Rep (2019) 9(1):13261. 10.1038/s41598-019-49860-0 31519967 PMC6744511

[128] Kanwar N Campion MB Schneider AR Milosevic D Sosa C Wojcik AA Validation and clinical utility of a pan-cancer circulating tumor DNA assay as a first-approach test. J Mol Diagn (2025) 27(12):1213–23. 10.1016/j.jmoldx.2025.08.009 41015180

[129] Dao J Conway PJ Subramani B Meyyappan D Russell S Mahadevan D . Using cfDNA and ctDNA as oncologic markers: a path to clinical validation. Int J Mol Sci (2023) 24(17):13219. 10.3390/IJMS241713219 37686024 PMC10487653

[130] Rompianesi G Martino MD Gordon-Weeks A Montalti R Troisi R . Liquid biopsy in cholangiocarcinoma: current status and future perspectives. World J Gastrointest Oncol (2021) 13(5):332–50. 10.4251/WJGO.V13.I5.332 34040697 PMC8131901

[131] Yu J He AR Ouf M Mehta R Anaya DA Denbo J Detecting early recurrence with circulating tumor DNA in stage I-III biliary tract cancer after curative resection. JCO Precis Oncol (2025) 9(9):e2400443. 10.1200/po-24-00443 39772829 PMC11723488

[132] Miura Y Ohyama H Mikata R Hirotsu Y Amemiya K Mochizuki H The efficacy of bile liquid biopsy in the diagnosis and treatment of biliary tract cancer. J Hepatobiliary Pancreat Sci (2024) 31(5):329–38. 10.1002/jhbp.1432 38523241

[133] Arechederra M Rullán M Amat I Oyon D Zabalza L Elizalde M Next-generation sequencing of bile cell-free DNA for the early detection of patients with malignant biliary strictures. Gut (2022) 71(6):1141–51. 10.1136/gutjnl-2021-325178 34285068 PMC9120390

[134] Berchuck JE Facchinetti F DiToro DF Baiev I Majeed U Reyes S The clinical landscape of cell-free DNA alterations in 1671 patients with advanced biliary tract cancer. Ann Oncol (2022) 33(12):1269–83. 10.1016/j.annonc.2022.09.150 36089135

[135] Quan X Huang X Liu J Yuan X Shu J . Preoperative assessment of longitudinal extent in hilar cholangiocarcinoma using noninvasive enhanced MR radiomics: a multicenter study. Front Oncol (2025) 15:1632630. 10.3389/fonc.2025.1632630 40978037 PMC12446029

[136] Cheng M Zhang H Guo Y Lyu P Yan J Liu Y Comparison of MRI and CT based deep learning radiomics analyses and their combination for diagnosing intrahepatic cholangiocarcinoma. Scientific Rep (2025) 15(1):9629. 10.1038/s41598-025-92263-7 40113926 PMC11926170

[137] Xu L Chen Z Zhu D Wang Y . The application status of Radiomics-Based machine learning in intrahepatic cholangiocarcinoma: systematic review and meta-analysis. J Med Internet Res (2025) 27(1):e69906. 10.2196/69906 40323647 PMC12089883

[138] Jang HJ Kim DH Choi SH Rhee H Cho ES Yeom SK Preoperative prediction of microvascular invasion in intrahepatic cholangiocarcinoma and its prognostic implications: a multicenter study. Liver Cancer (2026) 15(1):63–76. 10.1159/000547071 40709287 PMC12286615

[139] Miao G Qian X Zhang Y Hou K Wang F Xuan H An MRI-based radiomics model for preoperative prediction of microvascular invasion and outcome in intrahepatic cholangiocarcinoma. Eur J Radiol (2025) 183:111896. 10.1016/j.ejrad.2024.111896 39732135

[140] Fiz F Rossi N Langella S Ruzzenente A Serenari M Ardito F Radiomic analysis of intrahepatic cholangiocarcinoma: non-invasive prediction of pathology data: a multicenter study to develop a clinical–radiomic model. Cancers (2023) 15(17):4204. 10.3390/cancers15174204 37686480 PMC10486795

[141] Zhan PC Lyu PJ Li Z Liu X Wang HX Liu NN CT-Based radiomics analysis for noninvasive prediction of perineural invasion of perihilar cholangiocarcinoma. Front Oncol (2022) 12:900478. 10.3389/fonc.2022.900478 35795043 PMC9252420

[142] Zhan PC Yang T Zhang Y Liu KY Li Z Zhang YY Radiomics using CT images for preoperative prediction of lymph node metastasis in perihilar cholangiocarcinoma: a multi-centric study. Eur Radiol (2024) 34(2):1280–91. 10.1007/s00330-023-10108-1 37589900

[143] Pan YJ Wu S Zeng Y Cao ZR Shan Y Lin J Intra- and peri-tumoral radiomics based on dynamic contrast Enhanced-MRI to identify lymph node metastasis and prognosis in intrahepatic cholangiocarcinoma. J Magn Reson Imaging (2024) 60(6):2669–80. 10.1002/jmri.29390 38609076

[144] Qin H Hu X Zhang J Dai H He Y Zhao Z Machine-learning radiomics to predict early recurrence in perihilar cholangiocarcinoma after curative resection. Liver Int (2021) 41(4):837–50. 10.1111/liv.14763 33306240

[145] Song Y Zhou G Zhou Y Xu Y Zhang J Zhang K Artificial intelligence CT radiomics to predict early recurrence of intrahepatic cholangiocarcinoma: a multicenter study. Hepatol Int (2023) 17(4):1016–27. 10.1007/s12072-023-10487-z 36821045

[146] Fiz F Rossi N Langella S Conci S Serenari M Ardito F Radiomics of intrahepatic cholangiocarcinoma and peritumoral tissue predicts postoperative survival: development of a CT-Based clinical-radiomic model. Ann Surg Oncol (2024) 31(9):5604–14. 10.1245/s10434-024-15457-9 38797789

[147] Koku D Agarwala Y Vithayathil M Tait P Aboagye EO Sharma R . Radiomics of hepatopancreatobiliary cancer diagnosis, management, and future prospects. Clin Radiol (2026) 92:107167. 10.1016/j.crad.2025.107167 41365695

[148] Ronot M Calderado J . Incorporating artificial intelligence into imaging for surveillance and diagnosis of liver cancer: innovations, challenges, and clinical translation. Hepatology (2026). 10.1097/HEP.0000000000001698 41616271

[149] Dueland S Smedman TM Grut H Syversveen T Jørgensen LH Line PD . PET-uptake in liver metastases as method to predict tumor biological behavior in patients transplanted for colorectal liver metastases developing lung recurrence. Cancers (Basel) (2022) 14(20):5042. 10.3390/CANCERS14205042 36291826 PMC9599638

[150] Huang X Yang J Li J Xiong Y Abd-Elsalam S . Comparison of magnetic resonance imaging and 18-fludeoxyglucose positron emission tomography/computed tomography in the diagnostic accuracy of staging in patients with cholangiocarcinoma: a meta-analysis. Medicine (United States) (2020) 99(35):E20932. 10.1097/MD.0000000000020932 32871859 PMC7458197

[151] Lamarca A Barriuso J Chander A McNamara MG Hubner RA Óreilly D 18F-fluorodeoxyglucose positron emission tomography (18FDG-PET) for patients with biliary tract cancer: systematic review and meta-analysis. J Hepatol (2019) 71(1):115–29. 10.1016/j.jhep.2019.01.038 30797051

[152] Hu JH Tang JH Lin CH Chu YY Liu NJ . Preoperative staging of cholangiocarcinoma and biliary carcinoma using 18F-fluorodeoxyglucose positron emission tomography: a meta-analysis. J Invest Med (2018) 66(1):52–61. 10.1136/jim-2017-000472 28912249

[153] Pang L Bo X Wang J Wang C Wang Y Liu G Role of dual-time point 18F-FDG PET/CT imaging in the primary diagnosis and staging of hilar cholangiocarcinoma. Abdom Radiol (2021) 46(9):4138–47. 10.1007/s00261-021-03071-2 33825930

[154] Ma KW Cheung TT She WH Chok KSH Chan ACY Dai WC Diagnostic and prognostic role of 18-FDG PET/CT in the management of resectable biliary tract cancer. World J Surg (2018) 42(3):823–34. 10.1007/s00268-017-4192-3 28905105

[155] Hwang JP Moon JH Kim HK Lee MH Lim CH Park SB Prognostic value of metabolic parameters measured by pretreatment dual-time-point 18F-fluorodeoxyglucose positron emission tomography/computed tomography in patients with intrahepatic or perihilar cholangiocarcinoma A STROBE study. Medicine (United States) (2021) 100(21):e26015. 10.1097/MD.0000000000026015 34032720 PMC8154415

[156] Harimoto N Hoshino K Muranushi R Hagiwara K Yamanaka T Ishii N Impact of metabolic parameters of 18F-Fluorodeoxyglucose positron-emission tomography after hepatic resection in patients with intrahepatic cholangiocarcinoma. Anticancer Res (2019) 39(2):971–7. 10.21873/anticanres.13201 30711983

[157] Koca F Petrova E El Youzouri H Heil J Heise M Sliwinski S Prognostic value of resection margin and lymph node status in perihilar cholangiocarcinoma. HPB (2025) 27(1):71–9. 10.1016/j.hpb.2024.09.012 39455409

[158] Yin JX Fan X Chen QL Chen J He J . Progress in the application of fludeoxyglucose positron emission tomography computed tomography in biliary tract cancer. World J Hepatol (2025) 17(5):105446. 10.4254/WJH.V17.I5.105446 40501462 PMC12149906

[159] Cao J Srinivas-Rao S Mroueh N Anand R Kongboonvijit S Sertic M Cholangiocarcinoma imaging: from diagnosis to response assessment. Abdom Radiol (NY) (2024) 49(5):1699–715. 10.1007/S00261-024-04267-Y 38578323

[160] Pang L Mao W Zhang Y Liu G Hu P Chen S Comparison of 18F-FDG PET/MR and PET/CT for pretreatment TNM staging of hilar cholangiocarcinoma. Abdom Radiol (NY) (2023) 48(8):2537–46. 10.1007/S00261-023-03925-X 37179282

[161] Yoo J Lee JM Yoon JH Joo I Lee DH . Additional value of integrated 18F-FDG PET/MRI for evaluating biliary tract cancer: Comparison with contrast-enhanced CT. Korean J Radiol (2021) 22(5):714–24. 10.3348/kjr.2020.0689 33660461 PMC8076821

[162] Ferrone C Goyal L Qadan M Gervais D Sahani DV Zhu AX Management implications of fluorodeoxyglucose positron emission tomography/magnetic resonance in untreated intrahepatic cholangiocarcinoma. Eur J Nucl Med Mol Imaging (2020) 47(8):1871–84. 10.1007/S00259-019-04558-3 31705172

[163] Msherghi A Abuajamieh M Ekreer M Alzlitni M Hajalamin M Aldieb E Comparative diagnostic performance of [68 Ga]Ga-FAPI PET/CT and [18 F]FDGPET/CT in biliary tract cancers: a systematic review and meta-analysis. Eur J Nucl Med Mol Imaging. (2025) 52(11):4200–42. 10.1007/s00259-025-07264-5 40214739

[164] Zhang Z Cheng C Jiang H Pan G Yu Y Jin G 68Ga-FAPI-04 PET/CT for the evaluation of cholangiocarcinoma comparison with 18F-FDG PET/CT and abdominal 68Ga-FAPI-04 PET/MR. Clin Nucl Med (2024) 49(5):409–18. 10.1097/RLU.0000000000005112 38465929

[165] Pabst KM Trajkovic-Arsic M Cheung PFY Ballke S Steiger K Bartel T Superior tumor detection for 68Ga-FAPI-46 *versus* 18F-FDG PET/CT and conventional CT in patients with cholangiocarcinoma. J Nucl Med (2023) 64(7):1049–55. 10.2967/jnumed.122.265215 37024301 PMC10315700

[166] Pabst KM Weber MM Laschinsky C Sandach P Bartel T Küper AT [68Ga]Ga-FAPI-46 PET accuracy for cancer imaging with histopathology validation: a single-centre, single-arm, interventional, phase 2 trial. Lancet Oncol (2025) 26(9):1204–14. 10.1016/S1470-2045(25)00299-2 40774265

[167] Denizmen Zorba D Arslan DF Sanli Y Kuyumcu S . Optimizing Intrahepatic cholangiocarcinoma staging: comparison of [18F]FDG, [68Ga]Ga-FAPI, and [68Ga]Ga-Trivehexin PET/CT imaging. Clin Nucl Med (2026). 10.1097/RLU.0000000000006310 41627164

[168] Raeisi N Saber Tanha A Ghahraman M Aryana K Aghaee A . Comparable diagnostic value of 99mTc-FAPI-46 SPECT/CT and 18F-FDG PET/CT in cholangiocarcinoma: a case report. Clin Nucl Med (2025) 50:e695–e696. 10.1097/RLU.0000000000005877 40357631

[169] Gorji L Brown ZJ Limkemann A Schenk AD Pawlik TM . Liver transplant as a treatment of primary and secondary liver neoplasms. JAMA Surg (2024) 159(2):211–8. 10.1001/JAMASURG.2023.6083 38055245

[170] Dueland S Smedman TM Syversveen T Grut H Hagness M Line PD . Long-term survival, prognostic factors, and selection of patients with colorectal cancer for liver transplant: a nonrandomized controlled trial. JAMA Surg (2023) 158(9):e232932. 10.1001/JAMASURG.2023.2932 37494056 PMC10372758

[171] Soubrane O Scatton O . The development of transplant oncology may worsen the liver gap and needs new technical options in liver transplantation. Ann Surg (2023) 279(2):2023–4. 10.1097/sla.0000000000006086 37622319

[172] Cillo U Nalesso F Bertacco A Indraccolo S Gringeri E . Normothermic perfusion of a human tumoral liver for 17 days with concomitant extracorporeal blood purification therapy: case description. J Hepatol (2024) 81(3):e96–e98. 10.1016/j.jhep.2024.04.024 38703827

[173] Nalesso F Bertacco A Bettin E Cacciapuoti M Bogo M Cattarin L The rationale for combining normothermic liver machine perfusion with continuous renal replacement therapy to maintain physiological perfusate during *Ex Vivo* organ perfusion. J Clin Med (2024) 13(17):5214. 10.3390/jcm13175214 39274427 PMC11396463

[174] Cillo U Lauterio A Furlanetto A Canitano N Polacco M Buscemi V Full-left/full-right liver splitting with middle hepatic vein and caval partition during dual hypothermic oxygenated machine perfusion. Transplantation (2024) 108(6):1417–21. 10.1097/tp.0000000000005039 38755751

