# Supplementary material for: Present and future of liver transplantation for cholangiocellular carcinoma: moving toward personalized multiparametric transplantability patterns
Source: Transpl Int. 2026 May 7;39:15786. doi: 10.3389/ti.2026.15786 (PMC13189985; doi:10.3389/ti.2026.15786)
Supplement: Supplementary file 1 [file DataSheet1.pdf]

SUPPLEMENTARY MATERIALS

Supplementary Table 1: recommendations about liver transplantation for perihilar cholangiocarcinoma (pCCA)

Statement	Level of evidence (OCEBM)	Recommendation from guidelines	
**Hepatic resection is the main treatment for pCCA**	Low (level 4)[1–4]	**AASLD 2023[5]**	Surgical resection is the treatment of choice for early-stage pCCA and dCCA without any evidence of metastatic disease.
		**Milan Consensus 2024[6]**	Major hepatectomy along with resection of segment I and bile duct should be considered the standard approach to aim for R0 resection<br>(Reccomendation: Strong, [Level of Evidence: 4]
		**EASL 2025[7]**	Patients with localised pCCA should be treated with surgical resection if a complete resection (i.e. R0) is feasible with acceptable postoperative mortality<br>(LoE 2, strong recommendation, strong consensus).
**Gemcitabine-cisplatin-durvalumab is the standard of care systemic therapy for advanced biliary tract cancer**	High/very high (level 1-2)[8–12]	**AASLD 2023[5]**	Systemic chemotherapy is the first-line treatment of advanced CCA. Gem/cis is the standard of care for newly diagnosed patients.
		**Milan Consensus 2024[6]**	Patients with pCCA who have relapsed after curative surgery should be considered for chemotherapy (cisplatin-gemcitabine). The addition of immune-checkpoint inhibitors should be considered unless there are contraindications to immunotherapy. No current data is supporting the use of immune-checkpoint inhibitors in patients with severe autoimmune diseases such as Primary Sclerosing Cholangitis. Recommendation: Weak, [Level of Evidence: 5]
		**EASL 2025[7]**	Gemcitabine and cisplatin in combination with either durvalumab or pembrolizumab should be considered standard of care for the first-line systemic treatment of patients with unresectable or metastatic eCCA<br>(LoE 2, strong recommendation, consensus).
**LT plus chemo-radio therapy offers significant survival in selected patients with pCCA**	High/moderate (level 2/3)[13–17]	**Milan Consensus 2024[6]**	Patients diagnosed with pCCA should be considered for liver transplantation based on the following characteristics: Absence of general contraindications for transplantation, presence of an unresectable tumor (for anatomic consideration or due to underlying Primary Sclerosing Cholangitis) located above the cystic duct and 3.0 cm or less in its maximal radial diameter, with no evidence of nodal and distant metastases. Other exclusions are: attempted resection with violation of the tumor plane, positive transperitoneal biopsy of the primary tumor. Recommendation: Strong, [Level of Evidence: 3]<br><br>Patients should be treated with neoadjuvant therapy before liver transplantation. Recommendation: Strong, [Level of Evidence: 3]<br><br>Patients who undergo liver transplantation for pCCA should receive neoadjuvant chemo-radiotherapy with a total dose of 4500 cGy external beam radiotherapy with concomitant 5-FU infusion followed by a brachytherapy boost of 1500 cGy, followed by Capecitabine maintenance for at least 3-6 months before transplantation. Recommendation: Strong, [Level of Evidence: 2]

		**EASL 2025[7]**	Neoadjuvant chemoradiotherapy followed by LT can be considered for selected patients with early-stage (T1-2 which are less than 3 cm, N0, M0) unresectable pCCA (LoE 3, weak recommendation, strong consensus).
		**AASLD 2025[18]**	Patients with unresectable perihilar cholangiocarcinoma (CCA) that meet size criteria (< 3 cm in radial diameter) should be considered for liver transplant evaluation. (Strong, Level 3)<br><br>A neoadjuvant protocol with radiation and chemotherapy, and preoperative surgical staging in patients with perihilar CCA should be administered prior to transplantation. (Strong, Level 3)
**Is it possible to avoid neoadjuvant therapy before LT (concerns about the risk of vascular complications in particular after RT) in patients affected by pCCA**	Moderate (level 3)[16,17,19–21]	No specific statements.	
**In patients with PSC and pCCA is LT the treatment of choice?**	Moderate (level 3)[5,22,23]	**AASLD 2023[5]**	LT should be considered in all patients with PSC and complications of end-stage liver disease, recurrent cholangitis, intractable pruritus, or early-stage hepatobiliary cancers.<br><br>LT following neoadjuvant therapy should be considered for patients with pCCA (≤ 3 cm in radial diameter) that is unresectable or arising in the setting of PSC.
		**Milan Consensus 2024[6]**	Patients diagnosed with pCCA should be considered for liver transplantation based on the following characteristics: Absence of general contraindications for transplantation, presence of an unresectable tumor (for anatomic consideration or due to underlying Primary Sclerosing Cholangitis) located above the cystic duct and 3.0 cm or less in its maximal radial diameter, with no evidence of nodal and distant metastases. Other exclusions are: attempted resection with violation of the tumor plane, positive transperitoneal biopsy of the primary tumor.<br>Recommendation: Strong, [Level of Evidence: 3]
**Is it possible to consider LDLT for LT in pCCA patients? (concerns about vascular complications)**	Low/very low (level 4/5)[24–26]	**AASLD 2023[5]**	(*Alternatively, patients with PSC may benefit from receiving a living donor graft.)*
		**Milan Consensus 2024[6]**	In the setting of neoadjuvant chemoradiotherapy, both living donor and deceased donor liver transplantation may be considered for early stage, unresectable perihilar cholangiocarcinoma.<br>Recommendation: Weak, [Level of Evidence: 3]
**Is LT associated with superior outcomes against liver resection in resectable patients with pCCA?**	High (level 2)[15,16,27]	**Milan Consensus 2024[6]**	Liver transplantation may be considered instead of resection for resectable pCCA or when there is a question of resectability preoperatively, in the setting of appropriate access to living or deceased donors.<br>Recommendation: Weak, [Level of Evidence: 3]

Supplementary Table 2: recommendations about liver transplantation for intrahepatic cholangiocarcinoma (iCCA)

Statement	Level of evidence from cited litterature (OCEBM)	Recommendation from guidelines	
**Hepatic resection is the main treatment for iCCA.**	Low (level 4) [1,4,28–30]	**AASLD 2023[5]**	Surgical resection is the treatment of choice for patients with a single iCCA nodule in a resectable location without evidence of metastatic disease and who have adequate functional liver volume.
		**EASL-ILCA 2023[31]**	Resection of iCCA may be considered in selected patients with multifocal, unilobar iCCA (LoE 4, weak recommendation, consensus).
**Gemcitabine-cisplatin-durvalumab is the standard of care systemic therapy for advanced biliary tract cancer**	High/very high (level 1-2) [8–12,32]	**AASLD 2023[5]**	Systemic chemotherapy is the first-line treatment of advanced CCA. Gem/cis is the standard of care for newly diagnosed patients.
		**EASL-ILCA 2023[31]**	Patients with unresectable iCCA and good performance status should be treated with GemCis (as first-line chemotherapy), with the addition of durvalumab where available (LoE 1, strong recommendation, strong consensus).
**Is LT recommend for very early iCCA in cirrhotic patients?**	Moderate/low (level 3/4) [33–38]	**EASL-ILCA 2023[31]**	Liver transplantation for early stage iCCA (<3 cm) arising in the setting of cirrhosis can be considered, preferably under study protocols (LoE 4, weak recommendation, consensus)
		**AASLD 2023[5]**	LT for unresectable liver-limited iCCA should only be considered under research protocols.
		**ILTS-ILCA 2024[39]**	In cirrhotic patients with iCCA, liver transplantation may be considered as a potential therapeutic option in tumors ≤3 cm in diameter, after a period of observation with stability, and without extrahepatic metastasis, as it offers a chance of curative treatment and improved survival. (moderate level of evidence, moderate strength of recommendation).
		**AASLD 2025[18]**	Patients with biopsy-proven unresectable intrahepatic CCA or mixed-HCC-CCA with a maximum diameter < 3 cm, without extrahepatic disease, may be considered for LT. (Weak, Level 4)
**Is LT recommend for advanced iCCA in non cirrhotic patients?**	Moderate/low (level 3/4) [40–45]	**EASL-ILCA 2023[31]**	Liver transplantation for locally advanced iCCA should not be performed outside of clinical trials (LoE 4, weak recommendation, consensus).
		**AASLD 2023[5]**	LT for unresectable liver-limited iCCA should only be considered under research protocols.
		**ILTS-ILCA 2024[39]**	In non-cirrhotic patients with intrahepatic cholangiocarcinoma, liver transplantation is not routinely recommended but may be considered as part of investigational protocols for patients with unresectable, liver-confined disease after at least 6 months of stability after systemic therapy. Limitations on tumor size and number should be explored in prospective clinical trials. (moderate level of evidence, weak strength of recommendation).

**Is neoadjuvant therapy recommend before LT in iCCA patients**	Low (level 4) [42,43,45]	**AASLD 2023**[5]	*[…] Neoadjuvant therapy plus LT for patients with iCCA has also been evaluated in limited prospective case series with promising results. […]*
		**EASL-ILCA 2023**[31]	*[…] data on liver transplantation for locally advanced iCCA is scarce and the LoE is low. Patients with good and prolonged response to neoadjuvant chemotherapy may benefit from liver transplantation. […]*
		**ILTS-ILCA 2024**[39]	Bridging with neoadjuvant therapies, may help to identify patients with favorable tumor biology who might be considered for liver transplantation for unresectable iCCA (>6 months) (low level of evidence, weak strength of recommendation)
**Is LDLT an option for LT in iCCA patients?**	Low (level 4) [46–50]	**EASL-ILCA 2023**[31]	*[…] Consider the source of a donor liver and pre-transplant lymph node procurement […]*
		**ILTS-ILCA 2024**[39]	The available literature suggests that the indication for LDLT in iCCA could cautiously align with those for DDLT, particularly in very-early stage cases (low level of evidence, moderate strength of recommendation)
**Is LT associated with superior outcomes against liver resection in resectable patients with iCCA?**	Moderate/low (level 3/4) [51–58]	**AASLD 2023**[5]	LT for unresectable liver-limited iCCA should only be considered under research protocols.
		**EASL-ILCA 2023**[31]	*[…] Use of LT in iCCA and selection criteria for these patients is under active study. LT is considered for patients that are unresectable due to location, liver dysfunction, or bilobar disease […]*
		**ILTS-ILCA 2024**[39]	*[…] Transplantation is reserved for patients with unresectable iCCA lesions and favorable tumor biology. […]*

REFERENCES

1. Izquierdo-Sanchez L, Lamarca A, La Casta A, et al. Cholangiocarcinoma landscape in Europe: Diagnostic, prognostic and therapeutic insights from the ENSCCA Registry. *J Hepatol*. 2022;76(5):1109-1121. doi:10.1016/j.jhep.2021.12.010
2. Cillo U, Fondevila C, Donadon M, et al. Surgery for cholangiocarcinoma. *Liver Int*. 2019;39 Suppl 1(Suppl Suppl 1):143-155. doi:10.1111/liv.14089
3. Outcomes O, Expert F, Sugiura T, et al. Perihilar Cholangiocarcinoma – Novel Benchmark Values for Indicators for Outcome. 2021;274(5). doi:10.1097/SLA.0000000000005103
4. Valle JW, Kelley RK, Nervi B, Oh DY, Zhu AX. Biliary tract cancer. *The Lancet*. 2021;397(10272):428-444. doi:10.1016/S0140-6736(21)00153-7
5. Bowlus CL, Arrivé L, Bergquist A, et al. AASLD practice guidance on primary sclerosing cholangitis and cholangiocarcinoma. *Hepatology*. 2023;77(2):659-702. doi:10.1002/HEP.32771
6. Pfister M, Ratti F, Gores GJ, et al. Recommendations on Perihilar Cholangiocarcinoma. the Milan Jury-Based Consensus. *Ann Surg*. Published online 2025. doi:10.1097/SLA.0000000000006773
7. Marzioni M, Maroni L, Aabakken L, et al. EASL Clinical Practice Guidelines on the management of extrahepatic cholangiocarcinoma. *J Hepatol*. 2025;83(1):211-238. doi:10.1016/j.jhep.2025.03.007
8. Oh DY, Ruth He A, Qin S, et al. Durvalumab plus Gemcitabine and Cisplatin in Advanced Biliary Tract Cancer. *NEJM evidence*. 2022;1(8):EVIDoa2200015. doi:10.1056/EVIDoa2200015
9. Li Z, Aliseda D, Jones O, et al. Recent advances in systemic therapy for advanced biliary tract cancer: A systematic review and meta-analysis using reconstructed RCT survival data. *JHEP Reports*. 2025;7(3). doi:10.1016/j.jhepr.2024.101290
10. Oh DY, He AR, Bouattour M, et al. Durvalumab or placebo plus gemcitabine and cisplatin in participants with advanced biliary tract cancer (TOPAZ-1): updated overall survival from a randomised phase 3 study. *Lancet Gastroenterol Hepatol*. 2024;9(8):694-704. doi:10.1016/S2468-1253(24)00095-5
11. Kelley RK, Ueno M, Yoo C, et al. Pembrolizumab in combination with gemcitabine and cisplatin compared with gemcitabine and cisplatin alone for patients with advanced biliary tract cancer (KEYNOTE-966): a randomised, double-blind, placebo-controlled, phase 3 trial. *The Lancet*. 2023;401(10391):1853-1865. doi:10.1016/S0140-6736(23)00727-4
12. Goetze TO, Vogel A, Pratschke J, et al. Neoadjuvant chemotherapy with gemcitabine plus cisplatin followed by radical liver resection versus immediate radical liver resection alone followed adjuvant therapy in biliary tract cancer: Final results from the phase III AIO/CALGP/ACO-GAIN-Trial. *Journal of Clinical Oncology*. 2025;43(16_suppl):4008-4008. doi:10.1200/JCO.2025.43.16_SUPPL.4008
13. Darwish Murad S, Kim WR, Harnois DM, et al. Efficacy of neoadjuvant chemoradiation, followed by liver transplantation, for perihilar cholangiocarcinoma at 12 US centers. *Gastroenterology*. 2012;143(1):88-98.e3; quiz e14. doi:10.1053/j.gastro.2012.04.008
14. Cambridge WA, Fairfield C, Powell JJ, et al. Meta-analysis and Meta-regression of Survival after Liver Transplantation for Unresectable Perihilar Cholangiocarcinoma. *Ann Surg*. 2021;273(2):240-250. doi:10.1097/SLA.0000000000003801

15. Breuer E, Mueller M, Doyle MB, et al. Liver transplantation as a new standard of care in patients with perihilar cholangiocarcinoma? Results from an international benchmark study. *Ann Surg*. 2022;276(5):846-853. doi:10.1097/SLA.0000000000005641
16. Dong Y, Li Z, Podrascanin V, et al. Liver resection with and without vascular resection versus transplantation for de novo perihilar cholangiocarcinoma. *Hepatology*. Published online 2025. doi:10.1097/HEP.0000000000001449
17. Mantel HTJ, Westerkamp AC, Adam R, et al. Strict Selection Alone of Patients Undergoing Liver Transplantation for Hilar Cholangiocarcinoma Is Associated with Improved Survival. *PLoS One*. 2016;11(6):e0156127. doi:10.1371/journal.pone.0156127
18. Dove L, Chadha RM, Lai JC, et al. AASLD AST Practice Guideline on adult liver transplantation: Candidate evaluation. *Hepatology*. Published online December 17, 2025. doi:10.1097/hep.0000000000001644
19. Hoogwater FJH, Kuipers H, De Meijer VE, et al. Role of neoadjuvant chemoradiotherapy in liver transplantation for unresectable perihilar cholangiocarcinoma: multicentre, retrospective cohort study. *BJS Open*. 2023;7(2):1-8. doi:10.1093/bjsopen/zrad025
20. Mantel HTJ, Rosen CB, Helmbach JK, et al. Vascular complications after orthotopic liver transplantation after neoadjuvant therapy for hilar cholangiocarcinoma. *Liver Transplantation*. 2007;13(10):1372-1381. doi:10.1002/lt.21107
21. Gringeri E, Furlanetto A, Billato I, et al. The Italian experience on liver transplantation for unresectable peri - hilar cholangiocarcinoma : a national survey and future perspectives. *Updates Surg*. 2024;(0123456789). doi:10.1007/s13304-024-01889-1
22. Tan EK, Taner T, Heimbach JK, Gores GJ, Rosen CB. Liver Transplantation for Peri-hilar Cholangiocarcinoma. *J Gastrointest Surg*. 2020;24(11):2679-2685. doi:10.1007/s11605-020-04721-4
23. Jansson H, Olthof PB, Bergquist A, et al. Outcome after resection for perihilar cholangiocarcinoma in patients with primary sclerosing cholangitis: an international multicentre study. *HPB*. 2021;23(11):1751-1758. doi:10.1016/j.hpb.2021.04.011
24. Moon DB, Lee SG, Kim KH. Total hepatectomy, pancreatoduodenectomy, and living donor liver transplantation using innovative vascular reconstruction for unresectable cholangiocarcinoma. *Transplant International*. *Blackwell Publishing Ltd*. 2015;28(1):123-126. doi:10.1111/tri.12401
25. Ito T, Taura K, Fukumitsu K, et al. Safety and efficacy of living donor liver transplantation for unresectable perihilar cholangiocarcinoma: A single center prospective study. *J Hepatobiliary Pancreat Sci*. 2025;32(4):276-286. doi:10.1002/jhbp.12121
26. Tan EK, Rosen CB, Heimbach JK, Gores GJ, Zamora-Valdes D, Taner T. Living Donor Liver Transplantation for Perihilar Cholangiocarcinoma: Outcomes and Complications. *J Am Coll Surg*. 2020;231(1):98-110. doi:10.1016/j.jamcollsurg.2019.12.037
27. Moris D, Kostakis ID, Machairas N, et al. Comparison between liver transplantation and resection for hilar cholangiocarcinoma: A systematic review and meta-analysis. *PLoS One*. 2019;14(7):1-13. doi:10.1371/journal.pone.0220527
28. Alaimo L, Endo Y, Catalano G, et al. Benchmarks in Liver Resection for Intrahepatic Cholangiocarcinoma. *Ann Surg Oncol*. 2024;31(5):3043-3052. doi:10.1245/s10434-023-14880-8
29. Sposito C, Ratti F, Cucchetti A, et al. Survival benefit of adequate lymphadenectomy in patients undergoing liver resection for clinically node-negative intrahepatic cholangiocarcinoma. *J Hepatol*. 2023;78(2):356-363. doi:10.1016/j.jhep.2022.10.021
30. Mazzaferro V, Gorgen A, Roayaie S, Droz dit Busset M, Sapisochin G. Liver resection and transplantation for intrahepatic cholangiocarcinoma. *J Hepatol*. *Elsevier B.V.* 2020;72(2):364-377. doi:10.1016/j.jhep.2019.11.020

31. Alvaro D, Gores GJ, Walicki J, et al. EASL-ILCA Clinical Practice Guidelines on the management of intrahepatic cholangiocarcinoma. *J Hepatol*. 2023;79(1):181-208. doi:10.1016/j.jhep.2023.03.010
32. Wilbur HC, Soares HP, Azad NS. Neoadjuvant and adjuvant therapy for biliary tract cancer: Advances and limitations. *Hepatology*. Published online January 24, 2024. doi:10.1097/HEP.0000000000000760
33. Sapisochin G, De Lope CR, Gastaca M, et al. Intrahepatic cholangiocarcinoma or mixed hepatocellular-cholangiocarcinoma in patients undergoing liver transplantation: A spanish matched cohort multicenter study. *Ann Surg*. 2014;259(5):944-952. doi:10.1097/SLA.0000000000000494
34. Sapisochin G, Facciuto M, Rubbia-Brandt L, et al. Liver transplantation for "very early" intrahepatic cholangiocarcinoma: International retrospective study supporting a prospective assessment. *Hepatology*. 2016;64(4):1178-1188. doi:10.1002/hep.28744
35. Ziogas IA, Giannis D, Economopoulos KP, et al. Liver Transplantation for Intrahepatic Cholangiocarcinoma: A Meta-analysis and Meta-regression of Survival Rates. *Transplantation*. 2021;105(10):2263-2271. doi:10.1097/TP.0000000000003539
36. Tham EKJ, Lim RY, Koh B, et al. Prevalence of Chronic Liver Disease in Cholangiocarcinoma: A Meta-Analysis. *Clinical Gastroenterology and Hepatology*. 2025;23(10):1710-1718. doi:10.1016/J.CGH.2024.09.028
37. Thakral N, Gonzalez T, Nano O, Shin SH, Samuels S, Hussein A. Cirrhosis in intrahepatic cholangiocarcinoma: prognostic importance and impact on survival. *BMC Gastroenterol*. 2023;23(1):151. doi:10.1186/S12876-023-02710-W
38. De Martin E, Rayar M, Golse N, et al. Analysis of Liver Resection Versus Liver Transplantation on Outcome of Small Intrahepatic Cholangiocarcinoma and Combined Hepatocellular-Cholangiocarcinoma in the Setting of Cirrhosis. *Liver Transplantation*. 2020;26(6):785-798. doi:10.1002/lt.25737
39. Kodali S, Kulik L, D'Allessio A, et al. The 2024 ILTS-ILCA consensus recommendations for liver transplantation for HCC and intrahepatic cholangiocarcinoma. *Liver Transplantation*. 2025;31(6):815-831. doi:10.1097/LVT.0000000000000589
40. Hong JC, Jones CM, Duffy JP, et al. *Comparative Analysis of Resection and Liver Transplantation for Intrahepatic and Hilar Cholangiocarcinoma A 24-Year Experience in a Single Center*.
41. Hong JC, Petrowsky H, Kaldas FM, et al. Predictive index for tumor recurrence after liver transplantation for locally advanced intrahepatic and hilar cholangiocarcinoma. *J Am Coll Surg*. 2011;212(4):514-520. doi:10.1016/j.jamcollsurg.2010.12.005
42. Ito T, Butler JR, Noguchi D, et al. A 3-Decade, Single-Center Experience of Liver Transplantation for Cholangiocarcinoma: Impact of Era, Tumor Size, Location, and Neoadjuvant Therapy. *Liver Transpl*. 2022;28(3):386-396. doi:10.1002/LT.26285
43. Lunsford KE, Javle M, Heyne K, et al. Liver transplantation for locally advanced intrahepatic cholangiocarcinoma treated with neoadjuvant therapy: a prospective case-series. *Lancet Gastroenterol Hepatol*. 2018;3(5):337-348. doi:10.1016/S2468-1253(18)30045-1
44. Yaqub S, Busund S, Smedman TM, et al. Liver transplantation for locally advanced non-resectable intrahepatic cholangiocarcinoma treated with neoadjuvant therapy: early results from the TESLA trial. *British Journal of Surgery*. 2025;112(3). doi:10.1093/bjs/znaf054
45. McMillan RR, Javle M, Kodali S, et al. Survival following liver transplantation for locally advanced, unresectable intrahepatic cholangiocarcinoma. *American Journal of Transplantation*. 2022;22(3):823-832. doi:10.1111/ajt.16906

46. Kodali S, Connor AA, Thabet S, Brombosz EW, Ghobrial RM. Liver transplantation as an alternative for the treatment of intrahepatic cholangiocarcinoma: Past, present, and future directions. *Hepatobiliary and Pancreatic Diseases International. Elsevier (Singapore) Pte Ltd*. 2024;23(2):129-138. doi:10.1016/j.hbpd.2023.07.007
47. Rauchfuß F, Ali-Deeb A, Rohland O, Dondorf F, Ardelt M, Settmacher U. Living Donor Liver Transplantation for Intrahepatic Cholangiocarcinoma. *Curr Oncol*. 2022;29(3):1932-1938. doi:10.3390/curroncol29030157
48. Hara T, Eguchi S, Yoshizumi T, et al. Incidental intrahepatic cholangiocarcinoma in patients undergoing liver transplantation: A multi-center study in Japan. *J Hepatobiliary Pancreat Sci*. 2021;28(4):346-352. doi:10.1002/JHBP.896
49. Hafeez Bhatti AB, Tahir R, Qureshi NR, Mamoon N, Khan NY, Zia HH. Living donor liver transplantation for intra hepatic cholangiocarcinoma. *Annals of Medicine and Surgery*. 2020;57(June):82-84. doi:10.1016/j.amsu.2020.07.028
50. Sierra L, Barba R, Ferrigno B, et al. Living-Donor Liver Transplant and Improved Post-Transplant Survival in Patients with Primary Sclerosing Cholangitis. *J Clin Med*. 2023;12(8). doi:10.3390/JCM12082807
51. Pichlmayl R, Lamesch P, Weimann A, Tusch G, Ringe B. Surgical treatment of cholangiocellular carcinoma. *World J Surg*. 1995;19(1):83-88. doi:10.1007/BF00316984
52. Weimann A, Varnholt H, Schlitt HJ, et al. Retrospective analysis of prognostic factors after liver resection and transplantation for cholangiocellular carcinoma. *British Journal of Surgery*. 2000;87(9):1182-1187. doi:10.1046/j.1365-2168.2000.01532.x
53. Jung DH, Hwang S, Song GW, et al. Clinicopathological Features and Prognosis of Intrahepatic Cholangiocarcinoma After Liver Transplantation and Resection. *Ann Transplant*. 2017;22:42-52. doi:10.12659/AOT.901504
54. Hue JJ, Rocha FG, Ammori JB, et al. A comparison of surgical resection and liver transplantation in the treatment of intrahepatic cholangiocarcinoma in the era of modern chemotherapy: An analysis of the National Cancer Database. *J Surg Oncol*. 2021;123(4):949-956. doi:10.1002/jso.26370
55. Kim P, Littau M, Baker TB, et al. Intrahepatic cholangiocarcinoma: Is there a role for liver transplantation? *Surgery (United States)*. 2022;171(3):741-746. doi:10.1016/j.surg.2021.09.034
56. Lee Y Te, Singal AG, Lauzon M, et al. Disparities in curative treatments and outcomes for early stage intrahepatic cholangiocarcinoma in the United States. *Cancer*. 2022;128(20):3610-3619. doi:10.1002/cncr.34436
57. Howell TC, Rhodin KE, Shaw B, et al. Contemporary trends and outcomes after liver transplantation and resection for intrahepatic cholangiocarcinoma. *Journal of Gastrointestinal Surgery*. 2024;28(5):738-745. doi:10.1016/j.gassur.2024.02.029
58. Huang G, Song W, Zhang Y, Yu J, Lv Y, Liu K. Liver transplantation for intrahepatic cholangiocarcinoma: a propensity score-matched analysis. *Sci Rep*. 2023;13(1). doi:10.1038/s41598-023-37896-2
